# Volatile Compounds in *Citrus* Essential Oils: A Comprehensive Review

**DOI:** 10.3389/fpls.2019.00012

**Published:** 2019-02-05

**Authors:** M. Carmen González-Mas, José L. Rambla, M. Pilar López-Gresa, M. Amparo Blázquez, Antonio Granell

**Affiliations:** ^1^Departament de Farmacologia, Facultat de Farmàcia, Universitat de València, Valencia, Spain; ^2^Instituto de Biología Molecular y Celular de Plantas, Consejo Superior de Investigaciones Científicas – Universidad Politécnica de València, Valencia, Spain

**Keywords:** *citrus* essential oil, rind, flowers, leaves, volatile compounds, analytical methods

## Abstract

The essential oil fraction obtained from the rind of *Citrus* spp. is rich in chemical compounds of interest for the food and perfume industries, and therefore has been extensively studied during the last decades. In this manuscript, we provide a comprehensive review of the volatile composition of this oil fraction and rind extracts for the 10 most studied *Citrus* species: *C. sinensis* (sweet orange), *C. reticulata* (mandarin), *C. paradisi* (grapefruit), *C. grandis* (pummelo), *C. limon* (lemon), *C. medica* (citron), *C. aurantifolia* (lime), *C. aurantium* (bitter orange), *C. bergamia* (bergamot orange), and *C. junos* (yuzu). Forty-nine volatile organic compounds have been reported in all 10 species, most of them terpenoid (90%), although about half of the volatile compounds identified in *Citrus* peel are non-terpenoid. Over 400 volatiles of different chemical nature have been exclusively described in only one of these species and some of them could be useful as species biomarkers. A hierarchical cluster analysis based on volatile composition arranges these *Citrus* species in three clusters which essentially mirrors those obtained with genetic information. The first cluster is comprised by *C. reticulata, C. grandis, C. sinensis, C. paradisi* and *C. aurantium*, and is mainly characterized by the presence of a larger abundance of non-terpenoid ester and aldehyde compounds than in the other species reviewed. The second cluster is comprised by *C. junos*, *C. medica*, *C. aurantifolia*, and *C. bergamia*, and is characterized by the prevalence of mono- and sesquiterpene hydrocarbons. Finally, *C. limon* shows a particular volatile profile with some sulfur monoterpenoids and non-terpenoid esters and aldehydes as part of its main differential peculiarities. A systematic description of the rind volatile composition in each of the species is provided together with a general comparison with those in leaves and blossoms. Additionally, the most widely used techniques for the extraction and analysis of volatile *Citrus* compounds are also described.

## Introduction

*Citrus* essential oils, widely used to the procurance of natural fruity perfumes and as flavoring ingredients in food, pharmaceutical and cosmetic products (Tranchida et al., 2012; Palazzolo et al., 2013; Othman et al., 2017), are obtained mainly from the fruit rind (flavedo), although flowers or leaves have also been used. Volatile and semi-volatile compounds represent 85–99% of the entire oil fraction (Dugo and Mondello, 2011; Tranchida et al., 2012; Sarrou et al., 2013), which is represented typically by over 200 compounds. Hydrocarbon and derivate mono-and sesquiterpenes are the compounds most frequently reported, followed by aliphatic and olefinic C_6_–C_12_ non-terpene aldehydes, alcohols, ketones, esters and acids, along with several aromatic compounds. The non-volatile residue is mainly composed of flavonoids, coumarins, diterpenoids, sterols, and fatty acids.

Most of the studies regarding the composition of the volatile and semi-volatile fraction in *Citrus* ssp. show data from rind EOs (Dugo and Mondello, 2011). Besides, there are several papers reporting the volatile composition either from *neroli* (*Citrus* flowers EO) (Boussaada and Chemli, 2006; Boussaada et al., 2007; Sarrou et al., 2013; Družić, 2016), and *petitgrain* (*Citrus* leaves and little branches EO) (Lota et al., 1999, 2001a,b, 2002; Alonzo et al., 2000; Huang et al., 2000; Brophy et al., 2001; Vekiari et al., 2002, 2004; Fanciullino et al., 2005, 2006; Smadja et al., 2005; Boussaada and Chemli, 2006; Boussaada et al., 2007; Tomi et al., 2008; Singh et al., 2010; Guerrini et al., 2014; Družić, 2016; Paoli et al., 2016) or from extracts of *Citrus* flowers (Choi, 2003a; Flamini et al., 2007; Jabalpurwala et al., 2009; Flamini and Cioni, 2010; Cheong et al., 2011b) and leaves (Gancel et al., 2002, 2003, 2005).

The main objective of this review is to gather in a single manuscript the information on volatile and semi-volatile compounds that has been described up to date in rinds, flowers and leaves of the most studied *Citrus* species over the last two decades, when most of the work on *Citrus* essential oils has been performed. These species include *C. sinensis* (L.) Osb. (sweet orange), *C. reticulata* Bl. (mandarin), *C. paradisi* Macfad. (grapefruit), *C. grandis* (L.) Osb. (also *C. maxima* Burm.; pummelo), *C. limon* (L.) Burm. f. (lemon), *C. medica* L. (citron), *C. aurantifolia* (Christm.) Swingle (lime), *C. aurantium* L. (bitter orange), *C. bergamia* Risso et Poit. (bergamot orange) and *C. junos* Sieb. ex. Tanaka (yuzu), according to the classification of Tanaka (1954). Moreover, we used all the data of volatile compounds reviewed to perform a hierarchical clustering of these *Citrus* species based on the similarity of their rind volatile profiles. Additionally, the most widely used techniques for the extraction and analysis of volatile *Citrus* compounds are also described.

## Techniques to Extract *Citrus* Volatile Compounds

The EOs extraction from *Citrus* vegetal material (peel, flowers, and leaves) is based on steam distillation (Blanco Tirado et al., 1995), mainly in Clevenger-type hydrodistillation (Alonzo et al., 2000; Lota et al., 2001a,b; Vekiari et al., 2004; Boussaada and Chemli, 2006; Boussaada et al., 2007; Karioti et al., 2007; Darjazi, 2011a; Spadaro et al., 2012; Sarrou et al., 2013; Asikin et al., 2015; Fancello et al., 2016; Ben Hsouna et al., 2017). Recently, an improved Clevenger-type apparatus with a second condenser preventing thermal reactions and reducing the oxidation of some monoterpene compounds has been described (Chen et al., 2014). Moreover, EOs obtained from *Citrus* peel can also be performed by cold press extraction, whereas this technique should not be applied for *Citrus* flowers and leaves (Blanco Tirado et al., 1995; Merle et al., 2004; Choi, 2005, 2006; Dugo et al., 2005; Njoroge et al., 2005a,b; Sawamura et al., 2005; Asikin et al., 2012a,b; Tranchida et al., 2012; Sun et al., 2014b). The oil from *C. aurantium* (Dugo et al., 2011), *C. aurantifolia* (Craske et al., 2005) and specially *C. bergamia* (Poiana et al., 2003; Costa et al., 2010) peel obtained by cold pressing or solvent extraction, contains phototoxic furocoumarins of the bergapten type and it cannot be used neither in pharmaceutical nor in cosmetic industries. Then, Belsito et al. (2007) described a vacuum distillation which resulted in a high-quality *C. bergamia* essential oil, and therefore appropriate for commercial uses.

Other studies of *Citrus* peel composition use organic solvents such as pentane (Feger et al., 2001a; Miyazawa et al., 2010), pentane: ether (1:1) (Chisholm et al., 2003a,b), hexane (Inafuku-Teramoto et al., 2011; Miyazato et al., 2012, 2013), dichloromethane (Buettner et al., 2003; Craske et al., 2005; Cheong et al., 2011a; Omori et al., 2011; Cannon et al., 2015) or ethyl acetate (Jiang et al., 2011) to obtain different extracts. Also the organic system solvent *n*-pentane: diethyl ether (1:2) has been used to obtain the volatile organic compounds (VOCs) from *Citrus* flower (Alissandrakis et al., 2003). However, these extracts are not actually EOs, according to European Pharmacopeia (Spadaro et al., 2012). The EO should only be obtained either by distillation (water steam distillation or Clevenger hydrodistillation) or cold pressing. To prevent confusion, an organic extract from *Citrus* peel should not be named EO, (Alissandrakis et al., 2003; Chisholm et al., 2003b) although it could simply be named oil (Feger et al., 2001b; Buettner et al., 2003; Craske et al., 2005; Fisher et al., 2008). In fact, medium polarity solvents (diethyl ether, dichloromethane or ethyl acetate) extract more polar and higher MW compounds such as hexadecanal (Naef and Velluz, 2001; Gancel et al., 2002; Chisholm et al., 2003a,b; Cannon et al., 2015), squalene (Cheong et al., 2011b; Jiang et al., 2011), linoleic acid (Cheong et al., 2011b, 2012; Jiang et al., 2011), heptadecanoic acid (Delort and Jaquier, 2009; Jiang et al., 2011) or neophytadiene (Delort and Jaquier, 2009; Delort et al., 2015) than distillation or cold press extraction, and it fails to extract many monoterpene and sesquiterpene compounds which are characteristic of *Citrus* EOs (Jiang et al., 2011). Moreover, there are other minority extraction methods that use also organic solvents such as simultaneous distillation and extraction (SDE) (Akakabe et al., 2008; Kerdchoechuen et al., 2010; Sun et al., 2014a), microwave-assisted extraction (MAE) (Sun et al., 2014a; Liu et al., 2015), and ultrasonic-assisted extraction (UAE) (Alissandrakis et al., 2003; Darjazi, 2011b; Liu et al., 2012; Sun et al., 2014a; Zhang et al., 2017).

Additionally, several other extraction methods not involving the use of water or organic solvents have been employed. This is the case of water-free microwave extraction (Ferhat et al., 2007; Uysal et al., 2011); or supercritical CO_2_ fluid extraction (Akakabe et al., 2010; Dong et al., 2014; Sun et al., 2014a), a technique which extracts more efficiently higher MW compounds, such as squalene (Benelli et al., 2010) or linoleic acid (Dong et al., 2014). Headspace techniques have also been often used to extract volatile compounds, such as headspace extraction using a gas-washing bottle and a Porapak-Q sorbent tube (Elmaci and Onogur, 2012), and most frequently HS-SPME. HS-SPME has been used to extract volatiles and semi-volatile compounds of flowers and leaves, predominantly (Flamini et al., 2007; Jabalpurwala et al., 2009; Lin et al., 2010; Cheong et al., 2011b). When using this technique, the plant material is introduced in a septum-capped vial and after an equilibration period, a pre-conditioned fiber is exposed to the headspace of the vial for several minutes either at room temperature or at higher temperatures. There are currently several types of fibers suitable for adsorbing *Citrus* VOCs. A 100 μm PDMS-coated fiber was used to analyze the aroma of *C. unshiu* Marcov. fresh and dry blossoms (Choi, 2003a), *C. limon* flower organs and pollen (Flamini et al., 2007; Flamini and Cioni, 2010) and Taiwan *Citrus* leaves (Lin et al., 2010). In other studies, a 75 μm/85 μm CAR/PDMS-coated fiber was chosen to extract the VOCs from *Citrus* flowers (Jabalpurwala et al., 2009; Cheong et al., 2011b; Chung, 2012). Also, an additional two fibers have been used for *Citrus* peel oil. One fiber was a 65 μm DVB/PDMS coated fiber, which was used to analyze *C. clementine* EOs (González-Mas et al., 2015). For this analysis the oil was diluted 1:100 with dichloromethane and 10 μL were introduced in the septum-cap vial for HS-SPME with 990 μL of milli-Q water. Choi and Min (2004) also used this last fiber to analyze the VOCs in fresh flavedo of Hallabong *Citrus*. The other fiber used for *Citrus* oil and fresh flavedo was a 50 μm/30 μm DVB/CAR/PDMS fiber (Qiao et al., 2008; Mitropoulou et al., 2017), which is more polar than the other named fibers, and often used to extract volatile and semi-volatile compounds from the *Citrus* juices (González-Mas et al., 2011; Benjamin et al., 2013; Rambla et al., 2014; Qiu and Wang, 2015). Although recently it has also been used to analyze VOCs in *Citrus* flowers (Huang et al., 2017). According to the results reported in our literature review, the SPME-fibers used most often to analyze *Citrus* flower and leaf volatiles seems be the minor polar 100 μm PDMS or 75 μm/85 μm CAR/PDMS fibers, while the 65 μm DVB/PDMS fiber is more used for *Citrus* essential oil.

## Techniques Used to Analyze *Citrus* Volatile Compounds

The technique most widely used for the analysis of volatile and semi-volatile compounds in *Citrus* species currently is gas chromatography coupled to mass spectrometry (GC-MS). The principal option used to introduce the oil in the chromatograph is by direct injection of a dilution in organic solvent: most often pentane (Verzera et al., 2003; Boussaada and Chemli, 2006; Jazet Dongmo et al., 2009; Hosni et al., 2010) or hexane (Alonzo et al., 2000; Ahmed et al., 2001; Mondello et al., 2003; Dugo et al., 2005; Hamdan et al., 2010; Wang and Liu, 2014), but also dichloromethane (Blanco Tirado et al., 1995; Frizzo et al., 2004), diethyl ether (Merle et al., 2004) or even acetone (Viuda-Martos et al., 2009). Sun et al. (2014b) diluted the *C. maxima* EO in two steps: by 5,000-fold dilution in ethanol for analysis of the most abundant volatiles and 100-fold dilution for the minor volatiles. However, in other studies, the oil is not diluted (Mitiku et al., 2000; Song et al., 2000b; Choi et al., 2001; Minh Tu et al., 2002b, 2003b; Choi, 2005; Njoroge et al., 2005a,b, 2008; Sawamura et al., 2005; Akakabe et al., 2010; Cheong et al., 2011b; Omori et al., 2011). When volatile and semi-volatile compounds are extracted with an organic solvent, the samples are concentrated before direct injection using a rotary evaporator and further reduced under a stream of N_2_ (Hamdan et al., 2010; Miyazato et al., 2012). However, during these concentration steps, highly volatile compounds tend to be lost (Kerry et al., 2002). When VOCs are extracted with a fiber (HS-SPME), they are transferred directly to the injection port of the GC-MS system (Flamini et al., 2007; Jabalpurwala et al., 2009).

Different GC capillary columns have been used to analyze the volatile and semi-volatile *Citrus* compounds. Supplementary Table S1 in the Supporting Information shows the most often GC columns reported since 2010. Additionally, multidimensional gas chromatography consisting on the use of two columns in tandem with different separation characteristics (typically one polar and the other non-polar) has also been often used in the last decade, in order to achieve a higher chromatographic resolution of the diverse compounds comprising complex matrices such as EOs (Tranchida et al., 2012; Cannon et al., 2015).

There also exist more complex techniques based on carbon isotope ratios which allow the authentication of *Citrus* VOCs for the control of adulteration of *Citrus* essential oils. Thus, the employment of GC hyphenated to carbon isotope ratio mass spectrometry (GC-C-IRMS) can detect compounds of different botanical origin such as, for example, the presence of citral extracted from lemon grass (*Cymbopogon citratus*) (Schipilliti et al., 2018) in lemon EO. Moreover, this technique provides the authenticity assessment to differentiate natural and synthetic compounds in different *Citrus* essential oils, to discriminate *Citrus* peel oils of different geographical origin. Also, GC-C-IRMS is useful to eliminate the environmental influences and to differentiate genuine *Citrus* essential oils belonging to different species. However, this technique presents limitations related to chromatographic resolution due to instrumentation engineering. It will be necessary the optimization of the multidimensional GC separations to more effectively establish the ranges of authenticity of *Citrus* essential oils.

Despite the utility of GC-MS to analyze the volatile fractions of different biological samples, neither the profile of compounds identified by this technique nor the sensitivity in their detection correspond exactly with those the human nose perceives. To know the characteristic aroma of a *Citrus* oil or extract or fresh vegetal material, and to make an organoleptic evaluation of them, GC coupled to olfactometry (GC-O) should be used (Song et al., 2000a,b). This technique determinates the odor-threshold values of the volatile components eluted from the GC column and requires trained sniffers (panelists). It is well known that even though food products usually contain several hundreds of volatiles, most of them do not possess aroma activity in the existing concentration in the product. GC-O includes aroma extracts dilution analysis (AEDA) to identify the most potent odorants. For that, aroma extracts are successively diluted, and the compounds are separated by GC and sniffed. Results are then expressed as a FD factor. The FD factor of a particular odorant is the highest dilution at which it is detected. An odorant with a high FD factor can be judged as an important contributor to the characteristic flavor of that particular product. Comparatively, there are fewer studies about the *Citrus* volatile and semi-volatile fraction using GC-O as compared to GC-MS. Up to date, GC-O has been used for the analysis of peel oil of the following species: *C. sinensis* (Högnadóttir and Rouseff, 2003; Qiao et al., 2008), *C. reticulata* (Buettner et al., 2003; Chisholm et al., 2003a; Miyazaki et al., 2012), *C. grandis* (Cheong et al., 2011a; Chung et al., 2012; Li et al., 2016), *C. aurantium* (Song et al., 2000a), *C. paradisi* (Lin and Rouseff, 2001), *C. aurantifolia* (Chisholm et al., 2003b), *C. limon* (Cannon et al., 2015), *C. bergamia* (Sawamura et al., 2006), *C. junos* (Song et al., 2000b; Lan Phi et al., 2009; Miyazawa et al., 2009; Miyazato et al., 2012, 2013; Tomiyama et al., 2012), and the minor species *C. tamurana* Hort. ex Tanaka (Choi et al., 2001; Tao et al., 2014), *C. flaviculpus* Hort. ex Tanaka (Choi et al., 2002), *C. inflata* Hort. ex Tanaka (Minh Tu et al., 2003b), *C. natsudaidai* Hayata (Lan Phi et al., 2006), *C. kinokuni* Hort. ex Tanaka (kinokuni mandarin) (Miyazawa et al., 2010), *C. unshiu* Marcov (satsuma mandarin) (Miyazawa et al., 2010), *C. nobilis* Lauriro (wild *Citrus* Mangshanyegan) (Liu et al., 2012), *C. sudachi* Hort. ex Shirai (sudachi) (Tomiyama et al., 2012), *C. sphaerocarpa* Hort. ex Tanaka (kabosu) (Minh Tu et al., 2002b; Tomiyama et al., 2012), *C. jabara* Hort. ex Tanaka (Omori et al., 2011), *C. hystrix* D.C. (kaffir lime) (Jirapakkul et al., 2013), *Citrus* sp. (Kiyookadaidai) (Minh Tu et al., 2003a), *Citrus* hallabong (*C. unshiu* Marcov ×*C. sinensis* Osbeck) (Choi, 2003b), *C. nobilis* Lour. var. *microcarpa* Hassk. (Pontianak oranges) (Fisher et al., 2008; Dharmawan et al., 2009), *Fortunella japonica* Swingle (kumquat) (Choi, 2005) and the hybrid *C. aurantifolia* ×*Fortunella japonica* Swingle (Eustis limequat) (Casilli et al., 2014).

## Volatile Compounds Identified in Essential Oils From Peel, Leaves, and Flowers of *Citrus* Species

We have listed in this review over one thousand volatile and semi-volatile compounds that have been reported during the last two decades in rinds, leaves or flowers (EOs and solvent extracts) of *Citrus* species cultivated worldwide, although some early studies have also been included (Supplementary Table S2). In this review we have focused on the composition of *C. reticulata*, *C. grandis* (also named *C. maxima*), *C. sinensis*, *C. paradisi*, *C. aurantium*, *C. junos*, *C. medica*, *C. aurantifolia*, *C. bergamia*, and *C. limon*. This study also allowed us to establish which species are more similar or distant to each other according to the rind composition of their volatile and semi-volatile fractions as described in the literature. Finally, the volatile pattern of *Citrus* rinds has been compared to those of flowers and leaves.

Our literature review on VOCs in *Citrus* species corroborates some commonalities for the 10 most widely used and analyzed *Citrus* species. Thus, the EOs obtained from *Citrus* rinds always show limonene, a hydrocarbon monoterpene, as the most abundant compound, its concentration generally representing about 60–95% of the oil (Jing et al., 2014). However, limonene can show lower levels, as in *C. bergamia*, in which it can decrease down to 30% (Jing et al., 2014), or in *C. limon*, where it can decrease down to 48% (Lota et al., 2002). The following compounds in abundance are also monoterpenes, usually representing less than 15% (Supplementary Table S2), although γ-terpinene (Fanciullino et al., 2006) and linalyl acetate (Verzera et al., 2003) can reach an abundance of 23% and 36%, respectively. Non-terpenoid compounds very rarely represent more than 1%, although this does not necessarily mean that these compounds do not have an impact on the EO aroma. On the other hand, *Citrus* flowers and leaves show a different volatile profile. In these organs, limonene is not always the major volatile compound. Depending on the species, other monoterpenes such as linalool, β-myrcene or β-citronellol, in the case of flowers (Boussaada and Chemli, 2006; Jabalpurwala et al., 2009; Sarrou et al., 2013), or sabinene, in the case of leaves (Tomi et al., 2008), may be the most prominent compounds.

**FIGURE 1:**
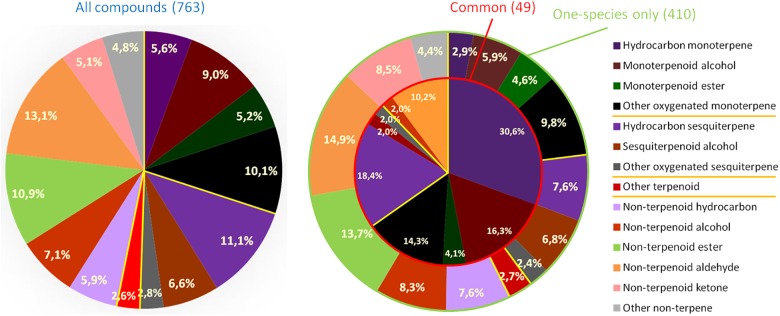
Relative frequency of each group of compounds present in the 10 most common *Citrus* species. *Left*, all the compounds described; *right outer circle*, compounds up to date detected in only one species (one-species only); *right inner circle*, compounds detected in all 10 species (common). Orange bars indicate separation between mono-, sesqui-, and other terpenoids, and non-terpenoid compounds. The qualitative data used is available in Supplementary Table S4.

**FIGURE 2:**
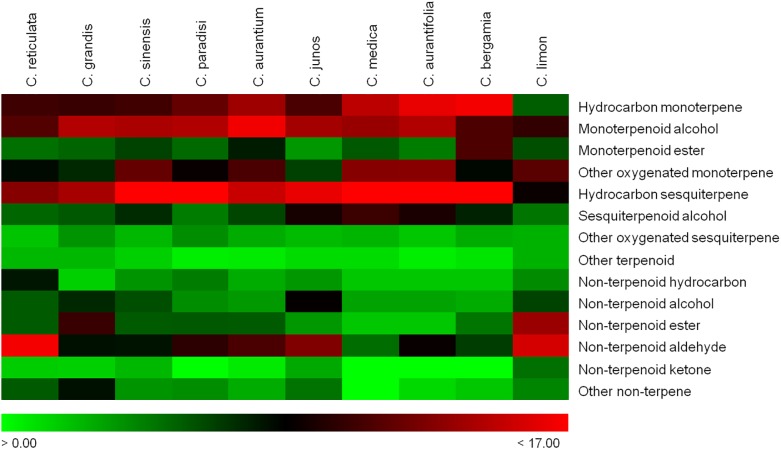
Heatmap showing the relative frequency of each group of compounds is presented for each of the most common *Citrus* species. Data represent the number of compounds reported in each category divided by the total number of compounds reported in each particular species, according to the scale below: *red*, high frequency; *black*, intermediate frequency; *green*, low frequency. The qualitative data used is available in Supplementary Table S5.

Attending to the number of compounds identified, sesquiterpene hydrocarbons are the most diverse group in most of the species. Monoterpene hydrocarbons and oxygenated monoterpene alcohols also tend to be among the most copious groups (Figure 1). Nevertheless, in some species such as *C. reticulata* or *C. limon*, the most numerous group corresponds to non-terpenoid aldehydes (Figure 2).

It became obvious when performing this review that the nomenclature of *Citrus* VOCs compounds, especially for mono- and sesquiterpene derivates needs standardization, as in its present form it can lead to misinterpretation of the results. Compounds are named according to IUPAC in some papers, but this nomenclature is very complex and results in very long names (Yu et al., 2009). Most of the papers use shorter non-systematic names, but this results in many synonyms for the same compound. An example of this is *p*-cymenene (Lota et al., 2002; Fanciullino et al., 2006; Pino et al., 2006), also named α-*p*-dimethylstyrene (Huang et al., 2000; Jabalpurwala et al., 2009; Cannon et al., 2015) and dehydro-4-cymene (Tomiyama et al., 2012). Similarly, (*Z*)-limonene oxide (Minh Tu et al., 2003b; Sun et al., 2014b) is also named (*Z*)-epoxylimonene (Blanco Tirado et al., 1995), (*Z*)-limonene,1,2-epoxide (Lota et al., 1999; Omori et al., 2011) or 1,2-epoxy-limonene (Casilli et al., 2014). Monoterpene compounds are named in many occasions according to the structure of *p*-menthane (Supplementary Figure S1A). This is the case of pseudolimonene (Lan Phi et al., 2009; Sun et al., 2014b), α-terpineol (Lota et al., 2002; Cheong et al., 2011b; Liu et al., 2012; Sun et al., 2014a), α-terpinyl acetate (Lota et al., 2002) (also α-terpenyl acetate) (Verzera et al., 2004), 4-terpinenol (Cheong et al., 2011b; Liu et al., 2012) and dihydrocarveol (Njoroge et al., 2005a) (also dehydrocarveol) (Shen et al., 2002), which have also been named as *p*-mentha-1(7),8-diene (Tomiyama et al., 2012), *p*-menth-1-en-8-ol (Chen et al., 2014), *p*-menth-1-en-8-yl acetate (Kirbaslar et al., 2001), 1-*p*-menthen-4-ol (Delort and Jaquier, 2009), and *p*-menth-8-en-2-ol (Delort et al., 2015), respectively. It seems necessary to reach a scientific agreement to name these compounds with a single criterion or, at the least, to indicate the several common synonyms for each compound in related articles. Along this review we have used one of the most common names for each compound, but all the different terms used in the scientific literature have been kept in Supplementary Table S2.

### Volatile Composition of Peel Extracts and Peel Essential Oil

**FIGURE 3:**
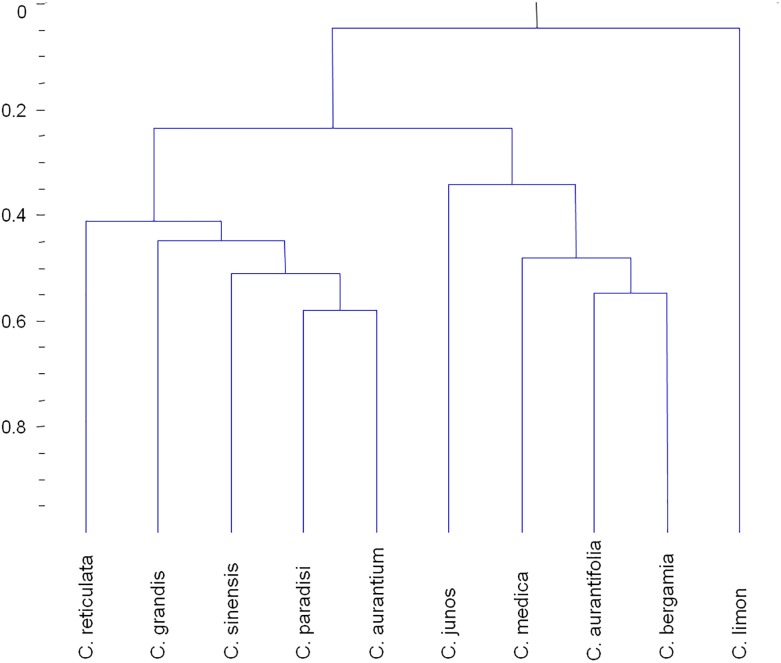
Hierarchical clustering of the 10 most common *Citrus* species according to their volatile profile as described in the scientific literature. For each species, data were normalized by dividing the number of times a particular compound was reported by the total number of reports of all the compounds in that species. The statistical parameters used were as described in Rambla et al. (2015). The qualitative data used to create HCA is available in Supplementary Table S4.

In this study, we have particularly focussed on the presence of volatile compounds in the peel of the 10 *Citrus* species most commonly analyzed, for which a significant amount of information is available in the scientific literature. Depending on each particular study, compounds have been identified with a different degree of confidence. We have systematically classified the reliability of identification for each compound in each of the revised articles in a scale from A+++ (highest confidence) to H (lowest confidence), as detailed in Supplementary Table S2. We considered it a key feature in this review, as our own experience in *Citrus* juice volatile profiling is that misidentification of volatile compounds is far to be rare in the scientific literature, particularly for those tentatively identified based on retention indices (González-Mas et al., 2011). Therefore, we have used a conservative approach all along this review for the description of the volatile profiles, as well as for the generation of figures and statistical analyses. We have selected only the 763 volatile and semi-volatile compounds which have been identified by, at least, both retention index and mass spectrum (reliability classified from A+++ to B in Supplementary Table S2), while discarding those identified with a lower degree of confidence. We used this approach to perform a qualitative description (described/never described) of the volatile profiles of these species, summarized in Figure 1, 2, and to elaborate the HCA shown in Figure 3. Applying this criterion, we observed that slightly more than half of these 763 reliably identified compounds are terpenoids, whilst the other half corresponds to a wide range of non-terpenoid compounds. Oxygenated monoterpenes is the most numerous group, comprising almost a quarter of all the compounds identified. Forty-nine of these volatile compounds have been described in all 10 species (Table 1 and Supplementary Figures S1B–H), almost 90% of which are terpenoid. Therefore, in some way they could be considered as those defining the characteristic *Citrus* volatile profile. Interestingly, most of these shared compounds are monoterpenoids (15 monoterpene hydrocarbons; 17 oxygenated monoterpenes) or sesquiterpenoids (9 sesquiterpene hydrocarbons; 2 oxygenated sesquiterpenes). The only non-terpenoid common volatiles are 1-octanol and the five C_8_ to C_12_ linear aliphatic aldehydes from octanal to dodecanal. On the other hand, 410 compounds of ample chemical diversity have been described up to date in only one of these species, most often in only one study (Figure 1 and Supplementary Table S2). It cannot be discarded that in some cases this might be due to misidentification, particularly when compound identification was not confirmed with a pure standard. Nevertheless, when unambiguously identified, they could be considered as biomarkers for each particular species.

**Table 1:** Common volatile compounds identified in *C. sinensis*, *C. reticulata*, *C. paradisi*, *C. grandis*, *C. aurantium*, *C. limon*, *C. medica*, *C. aurantifolia*, *C. bergamia*, and *C. junos*.

Groups of common compounds	Common compounds
Monoterpene hydrocarbons	β-Myrcene, (*Z*)-β-ocimene, (*E*)-β-ocimene, α-phellandrene, β-phellandrene, α-terpinene, γ-terpinene, limonene, terpinolene, sabinene, α-thujene, α-pinene, β-pinene, camphene, *p*-cymene
Monoterpenoid alcohols	linalool, (*Z*)-linalool oxide, β-citronellol, nerol, geraniol, terpinen-4-ol, α-terpineol, (*Z*)-sabinene hydrate
Monoterpenoid aldehydes	β-citronellal, neral, perillaldehyde
Other oxygenated monoterpenes	Citronellyl acetate, geranyl acetate, camphor, (*Z*)-limonene oxide, (*E*)-limonene oxide, carvone
Sesquiterpene hydrocarbons	(*E*,*E*)-α-farnesene, (*E*)-β-farnesene, β-elemene, δ-elemene, germacrene D, bicyclogermacrene, α-humulene, (*E*)-β-caryophyllene, δ-cadinene
Sesquiterpenoid alcohols	(*E*)-nerolidol
Other oxygenated sesquiterpenes	(*E*)-caryophyllene oxide
Non-terpenoid alcohols	1-octanol
Non-terpenoid aldehydes	Octanal, nonanal, decanal, undecanal, dodecanal


As previously described, several different -and somewhat complementary- analytical techniques have been used for the identification of the VOCs in the EOs along the scientific literature. In the case of these 10 species we considered that the number of studies already published is sufficient to rely on the total list of compounds identified as quite representative of their actual volatile profile. Therefore, we addressed the classification of these species by HCA based on their respective volatile profiles as described in the literature. This approach allowed us to classify these species into three clusters based on their volatile profile as described in the literature (Figure 3) (Rambla et al., 2015). The first cluster is comprised by *C. reticulata, C. grandis, C. sinensis, C. paradisi*, and *C. aurantium*, and is mainly characterized by the presence of a larger abundance of aliphatic and olefinic non-terpene compounds, principally esters and aldehydes (Figure 2). The second cluster is comprised by *C. junos*, *C. medica*, *C. aurantifolia*, and *C. bergamia*, and is characterized by the prevalence of mono- and sesquiterpene hydrocarbons. Finally, *C. limon* shows a particular volatile profile with some sulfur monoterpenoids and non-terpenoid esters and aldehydes as part of its main differential peculiarities (Figure 2, 3 and Supplementary Table S2).

This clustering shares some similarities with a recent phylogenetic analysis based on the chloroplast genomes of several *Citrus* species (Carbonell-Caballero et al., 2015). Most notably, *C. grandis*, *C. paradisi*, *C. sinensis*, and *C. aurantium* form a clade independent of another clade including *C. medica* and *C. aurantifolia* in both cluster analyses. Nevertheless, a remarkable difference between them is that *C. reticulata* is phylogenetically very different from the *C. sinensis* clade, whilst their volatile profiles are quite similar. Additionally, *C. limon* clustered with *C. grandis*, *C. paradisi*, *C. sinensis*, and *C. aurantium* in the chloroplast genome phylogenetic analysis, whilst it clusters separately from all these species when the volatile profile is considered.

However, a recent study about the origin and evolution of *Citrus* based on the total genome shows higher similarities with the volatile profiles here summarized. Our HCA clustered *C. sinensis*, *C. paradisi* and *C. aurantium* with *C. reticulata* and *C. grandis* (*C. maxima*). In fact, Wu et al. (2018) showed that the first three species are actually genetical hybrids and admixtures of the latter two. This genomic study also revealed that *C. aurantifolia* is a hybrid of *C. medica*. In accordance with this, our HCA based on the volatile profile of the fruit peel also clustered them together.

In the following sections, the volatile and semi-volatile profiles of the peel are presented independently, describing the peculiarities of each species. Species are presented following the results of the HCA, which provides information about the similarities and differences between them. The compounds up to date described in only one species have been highlighted (Supplementary Table S3), as well as those that allowed to establish correlations between species. In addition, the compounds quantitatively most abundant in each species have been indicated. Finally, the compounds frequently isolated in most *Citrus* but never reported in a particular species have also been mentioned here.

#### C. reticulata

Most of the VOCs identified in any of these *Citrus* species have also been isolated in *C. reticulata*. The EO of this species seems to differ principally from the others due to the presence of some exclusive non-terpenoid aldehyde compounds, such as (*E,E*)-2,4-heptadienal (Naef and Velluz, 2001) or (*Z*)-2-dodecenal (Naef and Velluz, 2001; Chisholm et al., 2003a) (Figure 2 and Supplementary Tables S2, S3). Moreover, several other non-terpenoid aldehyde compounds have been mainly isolated in the peel of *C. reticulata* and only sporadically in the peel of other *Citrus* species. This is the case of (*Z*)-4-decenal [*C. reticulata* (Naef and Velluz, 2001; Buettner et al., 2003; Chisholm et al., 2003a), *C. junos* (Miyazato et al., 2012) and *C. limon* (Cannon et al., 2015)], heptanal [*C. reticulata* (Dugo et al., 2005; Pino and Quijano-Celís, 2007; Zhang et al., 2017), *C. grandis* (Zhang et al., 2017), *C. sinensis* (Dugo and Mondello, 2011), and *C. limon* (Cannon et al., 2015)] or (*E*)-2-nonenal [*C. reticulata* (Naef and Velluz, 2001; Buettner et al., 2003; Pino and Quijano-Celís, 2007), *C. junos* (Miyazawa et al., 2009) and *C. limon* (Cannon et al., 2015)]. Although *C. limon* is the species with the volatile profile furthest from that of *C. reticulata*, also some non-terpenoid aldehyde compounds have only been reported so far in both *C. limon* (Cannon et al., 2015) and *C. reticulata* such as (*E*)-2-octenal (Naef and Velluz, 2001; Chisholm et al., 2003a), (*E,Z*)-2,4-nonadienal (Buettner et al., 2003), (2*E*,4*Z*,7*Z*)-decatrienal (Naef and Velluz, 2001), (*Z*)-5-dodecenal (Chisholm et al., 2003a) and (*Z*)-6-dodecenal (Ruberto and Rapisarda, 2002; Chisholm et al., 2003a). Among other compounds described in rinds of *C. reticulata*, we highlight the sesquiterpene hydrocarbon β-copaene, which has only been reported in the five species in this cluster according to their volatile profile [*C*. *reticulata* (Merle et al., 2004; Hosni et al., 2010; Luciardi et al., 2016), *C.* *sinensis* (Mondello et al., 2003; Hosni et al., 2010; Petretto et al., 2016), *C. paradisi* (Petretto et al., 2016), *C. aurantium* (Hosni et al., 2010), and *C. grandis* (Sawamura et al., 1991; Hosni et al., 2010)].

From a quantitative perspective, although non-terpenoid aldehyde compounds could help to differentiate this species from others, the most abundant compounds in *C. reticulata* EO are monoterpene hydrocarbons. Among them, the most relevant is limonene, usually representing about 95% of the total EO, but occasionally down to 60% in some studies (Fanciullino et al., 2006; Tao et al., 2014). The next compounds in abundance are γ-terpinene, sometimes reaching values above 15% (Mondello et al., 2003; Petretto et al., 2016), β-myrcene (7.43–0.1%) (Fanciullino et al., 2006; Tao et al., 2014), α-pinene (3.93–0.1%) (Fanciullino et al., 2006; Tao et al., 2014) or β-pinene (4%-traces) (Fanciullino et al., 2006). Monoterpenoids linalool and β-citronellal can reach up to 2.9% and 0.6%, respectively (Tao et al., 2014). Among other compounds with abundances approximately between 0.7 and 0.1% we found the sesquiterpene α-sinensal, the non-terpene aliphatic compounds octanal and decanal, and the aromatic compound methyl *N*-methylanthranilate (Supplementary Table S2). The rest of the compounds, including the non-terpenoid aldehydes, do not usually reach percentages higher than 0.1%, although this does not necessarily imply that they do not influence the final aroma of the EO.

#### C. grandis

The *C. grandis* peel is interesting as it presents several aromatic compounds that are almost exclusive for this species, such as indole [only described in *C. grandis* (Cheong et al., 2011a,b; Liu et al., 2012, 2015; Sun et al., 2014a,b; Li et al., 2016), *C. bergamia* (Verzera et al., 2003), and *C. limon* (Cannon et al., 2015)], or ethyl anthranilate (Cheong et al., 2011b), benzothiazole (Cheong et al., 2011a) (nitrogenous and sulfurous compound), 2-phenylethanol (Cheong et al., 2011b) [also in blossom and leaves of other *Citrus* species (Alissandrakis et al., 2003; Družić, 2016)], 3-ethylphenol (Cheong et al., 2011b), and methyl benzoate (Cheong et al., 2011a,b). Also, the non-terpenoid alcohols 1-hexanol and (*E*)-2-hexen-1-ol have been reported in the peel of *C. grandis* and other few *Citrus* species [*C. grandis* (Cheong et al., 2011a,b; Liu et al., 2012, 2015), *C. sinensis* (Högnadóttir and Rouseff, 2003; Liu et al., 2012), *C. reticulata* (Liu et al., 2012), *C. limon* (Cannon et al., 2015), and *C. junos* (Tomiyama et al., 2012)]. However, these compounds have been identified in a very low percentage, in many cases lower than 0.1%. The most abundant compounds are the same described in *C. reticulata*, also in very similar proportions. Only linalool has been usually identified with percentages lower than *C. reticulata*, close to 0.1% (Minh Tu et al., 2002a; Njoroge et al., 2005b; Cheong et al., 2011b), and γ-terpinene appears in this species between 0.05%-traces (Njoroge et al., 2005b; Cheong et al., 2011b). In any case, it is difficult to compare the results of different studies, because concentration of each compound is not always expressed in percentages; sometimes the authors express it in % w/w (Njoroge et al., 2008) or μg/g oil (Zhang et al., 2017). In addition, important variations in the VOCs concentration can occur within the same species, depending on cultivation conditions or varieties (Lota et al., 2002; Verzera et al., 2003) or even according to the type of oil extraction (Sun et al., 2014a).

The volatile profile most apart from *C. grandis* is that of *C. limon* (Figure 3). However, provided that *C. limon* has been widely studied by Cannon et al. (2015), several compounds have been exclusively isolated in *C. limon* and *C. grandis* peels, specially some non-terpenoid aliphatic ester compounds such as (*Z*)-3-hexenyl hexanoate (Sun et al., 2014a; Cannon et al., 2015) and methyl octanoate, methyl nonanoate and methyl decanoate (Cheong et al., 2011b; Cannon et al., 2015). Moreover, for this same reason, there are compounds only isolated in *C. grandis*, and *C. limon* and also *C. reticulata* or *C. sinensis* or *C. paradisi* such as (*E*)-piperitol [*C. grandis* (Sun et al., 2014a; Liu et al., 2015), *C. reticulata* (Liu et al., 2012), and *C. limon* (Cannon et al., 2015)].

One of the most relevant features of *C. grandis* volatile profile is the absence of some compounds that are typical of other common species of *Citrus*, mainly some sesquiterpene compounds. This is the case of the sesquiterpene (*E*)-α-bergamotene, which has never been reported in *C. grandis* peel but frequently identified in all the other common *Citrus* species studied (Mondello et al., 2003; Lan Phi et al., 2009; Aliberti et al., 2016; Petretto et al., 2016; Zhang et al., 2017). As far as we know, neither the compound thymol has been reported in *C. grandis* nor *C. paradisi* nor *C. bergamia* peel and neither (*E*)-2-decenal had been reported in *C. grandis* nor *C. aurantifolia*, but in the rest of the species reviewed.

Finally, it is important to indicate that when *Citrus* essential oils are exposed to sunlight, UV radiation or the air, the volatile profile can be modified due to chemical reactions. It has been reported in *C. grandis* oil that the concentration of several compounds, especially aldehydes such as decanal, dodecanal, neral or geranial, drops dramatically when exposed to these conditions. Instead, other compounds increase their concentration or appear novelly such as β-citronellal, α-humulene, linalool oxides, limonene oxides, (*E*)-carveol, (*Z*)-carveol, perilla alcohol, carvone, α-pinene oxide, γ-elemene or caryophyllene oxide (Supplementary Table S2) (Sun et al., 2014b; Li et al., 2016). This aldehyde transformation in *C. grandis* promoted an increase of strong oily notes and a decrease of fresh notes. Recently, sensory evaluation, GC-MS and GC-O demonstrated the pummelo rind oil co-treated by both oxygen and heating (70°C O_2_) showed significant differences in both volatile composition and aromatic profile from those of the corresponding fresh, nitrogen-protected (25°C N_2_), heated (70°C N_2_) and oxygen-exposed (25°C O_2_) oils (Sun et al., 2018). This study indicates that oxygen and heating co-treatment increases the content of limonene oxides and carvone in pummelo EO. The first compounds enhanced the sweet and floral notes, while the latter promoted the intensity of the minty note. These results indicate the importance of a correct production and storage of *Citrus* essential oil to preserve its original freshness quality.

#### C. sinensis

The volatile composition of *C. sinensis* peel EO is the most studied of all *Citrus* species along with those of *C. reticulata* and *C. limon* (Supplementary Table S2). This species seems to be richer in the diversity of sesquiterpene hydrocarbons than other species as *C. reticulata* or *C. limon*. An example of these compounds is aromadendrene (Njoroge et al., 2005a; Hosni et al., 2010) or sesquiphellandrene (Ruberto and Rapisarda, 2002; Sawamura et al., 2005), although it should not be forgotten that many of these sesquiterpenes are identified ambiguously, because there are no commercial standards or are unattainable for their price for many scientific studies. Also, in this species have been reported a relative large group of monoterpenoid compounds, being some of them almost exclusive compounds as limonene diepoxide (Högnadóttir and Rouseff, 2003; Njoroge et al., 2005a; Sawamura et al., 2005; Esquivel-Ferriño et al., 2014).

However, these distinctive VOCs of *C. sinensis* are minor compounds with a concentration usually lower than 0.1%. The major *C. sinensis* VOCs are very similar to that of *C. reticulata*, with comparable proportions. Limonene is usually reported between 90 and 97% in *C. sinensis*, although this percentage decreased down to 64% in some studies (Chen et al., 2014). Quantitatively, the most important difference with *C. reticulata* is for α-sinensal, since in *C. reticulata* this compound can reach a percentage above 0.7% (Lota et al., 2001b), while in *C. sinensis* its percentage does not rise of 0.05% (Njoroge et al., 2005a; Sawamura et al., 2005).

Recently the non-terpene aldehydes 2-butyl-2-octenal, 2-hexyl-2-decenal, and 2-octyl-2-dodecenal have each been detected in folded cold press orange oil at trace concentrations, confirmed with synthesized standards (Abreu et al., 2017). These compounds are hexanal, octanal, and decanal self-aldol condensation products, respectively. These self-aldol condensation products have attractive organoleptic qualities according to aroma and taste evaluations.

#### C. paradisi

The volatile profile from this species is very close to those of *C. aurantium* and *C. sinensis*, and a little less so to *C. grandis* and *C. reticulata* (Figure 3), and further apart from the other species studied. As in *C. sinensis*, numerous sesquiterpene hydrocarbons in have been reported in *C. paradisi* (Figure 2 and Supplementary Table S2), such as the sesquiterpene α-cedrene, isolated in *C. paradisi* (Njoroge et al., 2005b), *C. grandis* (Njoroge et al., 2005b), *C. sinensis* (Njoroge et al., 2005a; Sawamura et al., 2005), *C. aurantifolia* (Minh Tu et al., 2002a), and *C. medica* (Wu et al., 2013).

The major compounds in *C. paradisi* are very similar to those in *C. grandis*, often with lower levels of γ-terpinene and linalool to those with *C. sinensis* and *C. reticulata* (Njoroge et al., 2005b; Petretto et al., 2016), and with higher levels of nootkatone, which can reach 0.2% in *C. paradisi* (Njoroge et al., 2005b) and 177.02–5.05 μg/g in *C. grandis* (Zhang et al., 2017), while in the other two varieties they usually appear in lower percentages (Supplementary Table S2) (Ruberto et al., 1997; Zhang et al., 2017).

Again, although *C. limon* is the furthest variety of C. *paradisi*, there are compounds reported in *C. paradisi* and also in *C. limon* such as the compound undecanoic acid [*C. paradisi* (Lin and Rouseff, 2001; Njoroge et al., 2005b), *C. sinensi*s (Njoroge et al., 2005a; Sawamura et al., 2005), and *C. limon* (Cannon et al., 2015)].

#### C. aurantium

The volatile profile of *Citrus aurantium* (sour orange) is dominated by monoterpenoid compounds, such as isomenthol, geranyl propionate or eucalyptol (Figure 2) (Song et al., 2000a,b; Jabri Karoui and Marzouk, 2013). In fact, monoterpenoids comprise almost half of all the volatiles described in this species. Even so, the major compounds in *C. aurantium* are very similar to those of *C. sinensis* and *C. reticulata* (limonene, β-myrcene, β-pinene, linalool, (*E*)-β-ocimene, octanal) although in *C. aurantium* the percentages of linalyl acetate and geranyl acetate may reach 5% and 0.9%, respectively (Lota et al., 2001a; Petretto et al., 2016), while in *C. sinensis* and *C. reticulata* the percentages of these compounds do not usually exceed 0.1% (Mondello et al., 2003; Njoroge et al., 2005a, 2008). In addition, according to Jabri Karoui and Marzouk (2013), *C. aurantium* peel presents eucalyptol levels around 0.87%, significantly higher than those reported in other species. *C. aurantium* also seems to have higher levels of sesquiterpene germacrene D than in any other species (Lota et al., 2001a).

Some VOCS have been exclusively described in *Citrus aurantium*, principally monoterpene compounds, hydrocarbons such as α-ocimene (Baik et al., 2008) and oxygenated compounds such as α-terpinen-4-ol acetate (Pino and Rosado, 2000). The set of species most different to *C. aurantium* include *C. junos, C. bergamia, C. medica, C. aurantifolia* and especially *C. limon*, although some of them have several compounds in common with *C. aurantium* and some species in other clusters. This is the case of thymol, reported in *C. aurantium*, *C. reticulata*, *C. sinensis*, *C. aurantifolia*, *C. medica*, *C. junos* and *C. limon* (Supplementary Table S2) (Miyazawa et al., 2009; Dugo and Mondello, 2011; Aliberti et al., 2016; Zhang et al., 2017).

#### C. junos

According to the volatiles described in the peel, *C. junos* (yuzu) clusters with *C. medica* and *C. aurantifolia* and *C. bergamia*, although the similarity between their respective profiles is lower than those in the previous cluster (Figure 3). Its peel shows some exclusive VOCs, which could influence the aroma of its oil and peel extracts. In fact, in some cases, it has been proved by olfactometry that some of these compounds are strongly involved in the characteristic flavor of yuzu (Song et al., 2000b). These *C. junos* specific compounds belong to the group of sulfur compounds, aromatic compounds, terpenoid compounds, and non-terpenoid compounds of 7 to 12 carbons, most of which are also oxygenated, principally alcohols and aldehydes. These exclusive compounds are: methyltrisulfide (Song et al., 2000b) (odor characteristic component), 4-methyl-4-mercapto-pentan-2-one (Oh et al., 2007; Miyazawa et al., 2009), isoeugenol (Song et al., 2000b), the terpenoid compounds *p*-mentha-1,4,8-triene (Song et al., 2000b; Tomiyama et al., 2012), *p*-mentha-1(7),2-dien-8-ol (Tomiyama et al., 2012), geranyl butanoate (Song et al., 2000b), cedryl acetate (Song et al., 2000b), and δ-muurolene (Song et al., 2000b), (odor characteristic component), and the non-terpenoid hydrocarbon compounds (3*E*,5*Z*)-undeca-1,3,5-triene (Naef and Velluz, 2001; Miyazawa et al., 2009), (3*E*,5*Z*,8*Z*)-undeca-1,3,5,8-tetraene (Miyazawa et al., 2009; Miyazato et al., 2012), 6-methyl-5-hepten-2-ol (Song et al., 2000b) (odor characteristic component), (6*Z*,8*E*)-undeca-6,8,10-trien-3-ol (yuzuol) (Miyazawa et al., 2009; Tomiyama et al., 2012), (*E*)-6-nonenal (Miyazawa et al., 2009), (*E*)-4-decenal (Miyazawa et al., 2009), (*Z*)-4,5-epoxy-(*E*)-2-decenal (Miyazawa et al., 2009; Miyazato et al., 2012; Tomiyama et al., 2012), (*E*)-4,5-epoxy-(*E,Z*)-2,7-decadienal (Miyazato et al., 2012) (odor-active component), (*E*)-4-methyl-3-hexenoic acid (Miyazato et al., 2013) (odor-active component) and (6*Z*,8*E*)-undeca-6,8,10-trien-3-one (yuzunone) (Miyazawa et al., 2009; Miyazato et al., 2012; Tomiyama et al., 2012). It can be highlighted that some VOCs have only been reported so far in yuzu oil and in some of the other nine reviewed *Citrus* species (Supplementary Table S2). This is the case of the compound (*Z*)-9-dodecen-12-olide, also named yuzu lactone or oxacyclotridec-10-en-2-one, only isolated in *C. junos* (Miyazawa et al., 2009; Tomiyama et al., 2012) and *C. limon* (Cannon et al., 2015), or the compound decanoic acid, identified in *C. junos* (Song et al., 2000b; Tomiyama et al., 2012), *C. grandis* (Cheong et al., 2011b), *C. limon* (Cannon et al., 2015), *C. reticulata* (Pino and Quijano-Celís, 2007) and *C. aurantium* (Dugo and Mondello, 2011).

Most of the *C. junos* characteristic compounds previously mentioned are compounds that have been reported at trace levels, with percentages lower than 0.02%. In this species the major compounds are also very similar to those described in *C. sinensis* or *C. reticulata*. Thus, the most abundant compound remains limonene, although in concentrations between 63 and 77%, lower than usually those of the species closest to *C. reticulata* (Song et al., 2000b; Lan Phi et al., 2009). This decrease is associated to the increase of some monoterpene and sesquiterpene hydrocarbons, such as α-phellandrene, β-phellandrene, terpinolene or bicyclogermacrene (Song et al., 2000b; Lan Phi et al., 2009; Tomiyama et al., 2012).

It is important to emphasize that some VOCs frequently isolated in the other common *Citrus* species were not found in *C. junos* peel, such as geranial, isolated in the peel of all the other nine *Citrus* species, terpenyl acetate, neryl acetate, eucalyptol (also missing in *C. medica* peel), tetradecanal, β-bisabolene or decyl acetate (the latter two compounds found in the juice but not in the peel of C. *junos*) (Tomiyama et al., 2012).

#### C. medica

According to our review, the volatile profile most similar to that of *C. medica* are those of *C. aurantifolia* and *C. bergamia*, whilst C. *junos* also clusters together in terms of volatile profile similarity (Figure 3). As in *C. bergamia*, *C. aurantifolia* and *C. junos* peel, and also in *C. sinensis* and *C. paradisi*, many sesquiterpene hydrocarbons have been described in *C. medica* (Figure 2). The recent study realized by Aliberti et al. (2016) has reported some compounds in *C. medica* not described, or very rarely identified, in other *Citrus* species so far as like the monoterpenes cuminyl alcohol, dehydrosabina ketone or *cis*-4-caranone, and specially sesquiterpene like longifolene, 9-epi-caryophyllene, α-cuprenene, γ-cuprenene, italicene, nootkatol, or β-oplopenone, among many others. However, it is important to note that most of these compounds were tentatively identified, because a standard was not used for their identification, with some exception like the monoterpene *trans*-4-caranone and the sesquiterpenes longifolene or 9-epi-caryophyllene.

As in all the revised species, the most abundant compound in *C. medica* is limonene, but its percentage can decrease down to 51% (Verzera et al., 2005), while other monoterpene compounds such as γ-terpinene, β-pinene or camphene are present at higher concentration, in comparison with species of *C. reticulata* cluster where these compounds are usually described in percentages below 1%. Thus, in *C. medica* oil, γ-terpinene, β-pinene or camphene can reach percentage of 31%, 9.7%, and 10%, respectively (Lota et al., 1999; Aliberti et al., 2016; Petretto et al., 2016). Also *C. medica* presents higher concentrations of some sesquiterpenes, as is the case of (*E*)-α-bergamotene (Aliberti et al., 2016) or germacrene D (Petretto et al., 2016), although their abundance is usually lower than 0.5%.

Finally, *C. medica* peel lacks some compounds especially non-terpenoid aldehyde that many times are also absent in *C. aurantifolia* and/or *C. bergamia* peel, but which have been detected in the rind of all the other species analyzed. Thus, (*E*,*E*)-2,4-decadienal has never been reported in *C. medica* and *C. bergamia*, and (*Z*)-3-hexenal, (*E*)-2-dodecenal and the sesquiterpenoid α-sinensal have not been reported in *C. medica*, *C. aurantifolia* and *C. bergamia*. As far as we know, other compounds which have not been reported in *C. medica* peel are: (*E*)-2-hexenal, neither reported in *C. aurantium* nor in *C. bergamia* peel, or hexanal, neither present in *C. bergamia*, *C. aurantium* nor in *C. junos* peels. Also, the aromatic monoterpenoid carvacrol has not been described in *C. medica*, *C. bergamia*, *C. aurantifolia* or *C. paradisi*.

#### C. aurantifolia

Many mono- and sesquiterpene hydrocarbons and oxygenated monoterpenes have been reported in *C. aurantifolia* peel (Figure 2). In fact, *C. aurantifolia* has some exclusive terpenes such as the sesquiterpene santal-10-en-2-ol (Lota et al., 2002) (Supplementary Tables S2, S3). According to our cluster, the species most similar to *C. aurantifolia* is *C. bergamia* and further are *C. medica* and *C. junos* (Figure 3).

Despite *C. sinensis*, *C. reticulata*, *C. paradisi, C. grandis* and *C. aurantium* and specially *C. limon* clustering further away from *C. aurantifolia*, some terpenes are present in *C. aurantifolia* peel and in some of these other species, such as the oxygenated monoterpene fenchol, only isolated in *C. aurantifolia* (Selvaraj et al., 2002; Chisholm et al., 2003b; Fouad and da Camara, 2017), *C. grandis* (Cheong et al., 2011a) and *C. limon* (Ferhat et al., 2007) peels (Figure 3 and Supplementary Table S2). Despite the distance with *C. limon*, there are some terpene compounds which have been exclusively reported in *C. aurantifolia* and *C. limon*, such as the monoterpene geranic oxide [*C. aurantifolia* (Chisholm et al., 2003b) and *C. limon* (Cannon et al., 2015)]. Interestingly, the monoterpenoid isogeranial and the sesquiterpene α-santalene have been described only in *C. aurantifolia* (Feger et al., 2000; Costa et al., 2014), *C. medica* (Venturini et al., 2014; Mitropoulou et al., 2017) and *C. limon* (Vekiari et al., 2002; Cannon et al., 2015).

As in *C. medica*, the percentage of limonene may drop to 39.9% in the oil of *C. aurantifolia* (Lota et al., 2002), and the abundance of other terpene compounds is increased, such as β-pinene, neryl acetate, geranyl acetate, β-bisabolene, (*E*)-α-bergamotene, germacrene D and β-caryophyllene (Lota et al., 2002; Minh Tu et al., 2002a), similarly to what occurs in *C. medica*.

Despite *C. aurantifolia* peel being rich in total terpenes, it is striking that we do not find some terpene compounds found in other *Citrus* species, as indicated in the previous section about *C. medica*. Among the terpenes absent in *C. aurantifolia*, that were not mentioned above, we highlight the sesquiterpenoid nootkatone, also not reported in *C. junos*.

#### C. bergamia

As in *C. aurantifolia* and in *C. medica* peel, copious mono- and sesquiterpene hydrocarbons have been reported in *C. bergamia* (Figure 2 and Supplementary Table S2). Thus, several sesquiterpenoid compounds are principally isolated in *C. bergamia*, and in its more related species and in *C. limon*. A notable example is the sesquiterpene α-bisabolol, frequently isolated in *C. bergamia* (Sawamura et al., 2006; Belsito et al., 2007; Costa et al., 2010; Furneri et al., 2012), *C. limon* (Blázquez and Carbó, 2015; Cannon et al., 2015; Loizzo et al., 2016; Zhang et al., 2017), *C. aurantifolia* (Gancel et al., 2002; Minh Tu et al., 2002a; Chisholm et al., 2003b; Zhang et al., 2017), and *C. medica* (Lota et al., 1999; Verzera et al., 2005; Aliberti et al., 2016). Even there are terpene compounds that have been reported in *C. bergamia* and just once in *C. limon* as the monoterpene linalyl propionate [*C. bergamia* (Poiana et al., 2003; Verzera et al., 2003; Costa et al., 2010) and *C. limon* (Cannon et al., 2015)]. Also, several hydrocarbon and also oxygenated monoterpenes such as δ-terpinene (Poiana et al., 2003), (*Z*)-sabinene hydrate acetate (Poiana et al., 2003), and (*E*)-sabinene hydrate acetate (Verzera et al., 2003), seems exclusive of *C. bergamia* (Supplementary Table S2). In contrast, the sesquiterpenes α-copaene and valencene have not been found in *C. bergamia*, although it has been identified in all the other species studied (Song et al., 2000b; Zhang et al., 2017), excepting valencene, neither reported in *C. junos*.

The major compound in *C. bergamia* is limonene, but its percentage can decrease down to 33% (Poiana et al., 2003). Instead, *C. bergamia* shows higher percentage of other monoterpene compounds, most notably linalyl acetate, which is sometimes almost as abundant as limonene, which does not happen for any other species of the reviewed, as far as we know (Poiana et al., 2003; Verzera et al., 2003). Linalool is also more abundant in *C. bergamia* EO than in any other of these species; it can reach up to 13.9% in SC-CO_2_ extracts (Poiana et al., 2003). Moreover, other terpenes which are abundant in *C. medica* and *C. aurantifolia* are also increased in *C. bergamia*, when compared to the species of the *C. reticulata* cluster, such as α-pinene, β-pinene, neryl acetate, geranyl acetate, β-bisabolene, (*E*)-α-bergamotene, β-caryophyllene (Mondello et al., 2003; Poiana et al., 2003; Verzera et al., 2003; Sawamura et al., 2006; Belsito et al., 2007). Finally, compounds such as octyl acetate are more abundant in *C. bergamia* than in other species, while compounds such as octanal seems to decrease, with values lower than 0.1% (Poiana et al., 2003; Verzera et al., 2003).

On the other hand, several coumarin and psoralen (furocoumarin) compounds have been isolated in *C. bergamia* EOs. These compounds are more polar and less volatile than most of the compounds in *Citrus* EOs. Not surprisingly, they has been mainly reported in oils obtained by cold press (Costa et al., 2010; Russo et al., 2015), organic solvent (polar extracts) (Chisholm et al., 2003b; Craske et al., 2005), or more recently by ultrasonic (Zhang et al., 2017) extractions, and are usually identified by means of HPLC techniques (Costa et al., 2010; Russo et al., 2015). In some studies, such compounds are considered as part of the non-volatile peel fraction (Costa et al., 2010). Following this criterion, we have not included them as part of the volatile profile, although some of them have been listed in Supplementary Table S2. Although this type of compounds are usually not included in *Citrus* peel oil studies, when reported they have been principally described in *C. bergamia, C. aurantifolia* and *C. aurantium* EOs, and also in other oils such as those from *C. limon* (Bonaccorsi et al., 1999; Cannon et al., 2015; Russo et al., 2015). These compounds have not been found in *C. sinensis* or *C. reticulata* peel, which instead are the richest in polymethoxyflavones such as tangeretin (Bonaccorsi et al., 1999; Russo et al., 2015). Among them, the furocoumarins bergaptene (Poiana et al., 2003; Belsito et al., 2007; Costa et al., 2010; Russo et al., 2015) and bergamottin (Costa et al., 2010; Russo et al., 2015) are characteristic of the rind of *C. bergamia*, but they are not exclusive for this species as they have also been described in other species as *C. aurantifolia* (Craske et al., 2005; Russo et al., 2015), *C. limon* (only bergamottin) (Bonaccorsi et al., 1999; Russo et al., 2015), *C. paradisi* (Russo et al., 2015), and *C. aurantium* (Dugo et al., 1996, 2011; Pino and Rosado, 2000; Russo et al., 2015). Other examples are citropten [*C. aurantifolia* (Craske et al., 2005; Russo et al., 2015; Zhang et al., 2017), *C. bergamia*, and *C. limon*] or 5-geranyloxy-7-methoxy-coumarin [*C. aurantifolia* (Russo et al., 2015), *C. bergamia* and *C. limon*].

#### C. limon

Lemon EO is the most different of the commonly studied *Citrus* species in terms of volatiles. The volatile profile of *C. limon* has been analyzed in numerous studies (Dugo and Mondello, 2011; Liu et al., 2012; Spadaro et al., 2012; Cannon et al., 2015; Zhang et al., 2017). As far as we could find in our review mining, the majority of the volatile and semi-volatile compounds found in any other of these 10 *Citrus* species have also been described in *C. limon*. Moreover, Cannon et al. (2015) identified over 150 compounds by dichloromethane extraction in *C. limon* peel, which we could not find in any other study on VOCs in *Citrus*. Among them, we highlight those identified without ambiguity such as sulfur monoterpenoid compounds 2-(5-isopropyl-2-methyltetrahydrothiophen-2-yl)ethanol, 3-mercapto-3,7-dimethyl-6-octenyl acetate (odor-active component), 2-(5-isopropyl-2-methyltetrahydrothiophen-2-yl) ethyl acetate, 2-(5-isopropylidene-2-methyltetrahydrothiophen-2-yl) ethyl acetate and 2-[5-(1-hydroxy-1-methylethyl)2-methyltetrahydrothiophen-2-yl] ethyl acetate; other two sulfur non-terpenoid compounds, 2-propanoylthiophene and 2-(2-methyltetrahydrothiophen-2-yl) ethyl acetate; and the branched-chain aliphatic aldehydes 6-methylnonanal, 4-methyldecanal, 6-methyldecanal, 3-methylundecanal, 4-methylundecanal, 3-methyldodecanal, 4-methyldodecanal, and 4-methyltridecanal. All these exclusive compounds were identified as traces.

Quantitatively, the major compound of *C. limon* EO is limonene, at levels usually ranging between 70 and 48%, similar to those in *C. medica* and *C. aurantifolia*, and lower than those in the species comprising the first cluster. The following more abundant compounds are terpenoids similar to those higher in *C. medica* and *C. aurantifolia*. Among these compounds which have not been named so far, we can highlight geranial and neral, that in *C. limon* (Lota et al., 2002; Loizzo et al., 2016) can reach abundances of 2.9% and 1.5%, respectively, compared to percentages between 0.3%-traces in the species in the *C. reticulata* cluster (Minh Tu et al., 2002a; Ruberto and Rapisarda, 2002; Mondello et al., 2003; Tao et al., 2014).

It is also important to note that there is a set of sesquiterpene compounds that have been very frequently identified in *C. limon* peel, and with relative high frequency also in *C. aurantifolia* and *C. bergamia*. These compounds seem to form a small subgroup that characterizes these three species, although some of them may sporadically be found in other *Citrus* common species, such as *C. medica*. This is the case of the previously named α-bisabolol, and also of (*E*)-α-bisabolene, which has been very frequently isolated in *C. limon* (Verzera et al., 2001; Lota et al., 2002; Liu et al., 2012; Spadaro et al., 2012; Blázquez and Carbó, 2015; Cannon et al., 2015; Petretto et al., 2016), in *C. aurantifolia* (Feger et al., 2000; Craske et al., 2005; Costa et al., 2014) and occasionally in *C. paradisi* (Flamini and Cioni, 2010), *C. medica* (Lota et al., 1999) and *C. bergamia* (Costa et al., 2010); (*Z*)-α-bisabolene, very frequently isolated in *C. limon* (Verzera et al., 2001; Ferhat et al., 2007; Spadaro et al., 2012; Blázquez and Carbó, 2015; Cannon et al., 2015; Zhang et al., 2017) and *C. bergamia* (Verzera et al., 2003; Cosimi et al., 2009; Costa et al., 2010), and occasionally in *C. aurantifolia* (Feger et al., 2000; Zhang et al., 2017), *C. medica* (Verzera et al., 2005), and *C. aurantium* (Lota et al., 2001a); γ-curcumene, frequently reported in *C. limon* (Ferhat et al., 2007; Blázquez and Carbó, 2015; Cannon et al., 2015; Petretto et al., 2016), *C. bergamia* (Costa et al., 2010) and *C. aurantifolia* (Feger et al., 2000; Costa et al., 2014); β-santalene, isolated mainly in *C. limon* (Spadaro et al., 2012; Blázquez and Carbó, 2015; Cannon et al., 2015; Petretto et al., 2016), *C. bergamia* (Verzera et al., 2003; Cosimi et al., 2009; Costa et al., 2010; Furneri et al., 2012), and *C. aurantifolia* (Feger et al., 2000; Minh Tu et al., 2002a; Costa et al., 2014; Zhang et al., 2017); and campherenol, reported mainly in *C. limon* (Verzera et al., 2004; Spadaro et al., 2012; Cannon et al., 2015) and *C. bergamia* (Mondello et al., 2003; Verzera et al., 2003; Costa et al., 2010; Furneri et al., 2012), and sporadically in *C. aurantifolia* (Dugo and Mondello, 2011) and *C. medica* (Lamine et al., 2018). Unfortunately, the identification of most of these sesquiterpenes is not totally reliable, since it seems that standards of these compounds are not commercially available. Other compound with a similar pattern is the norcarotenoid 6-methyl-5-hepten-2-one, frequently reported in *C. limon* (Lota et al., 2002; Verzera et al., 2004; Spadaro et al., 2012; Blázquez and Carbó, 2015; Cannon et al., 2015) and *C. bergamia* (Mondello et al., 2003; Verzera et al., 2003; Sawamura et al., 2006; Cosimi et al., 2009; Costa et al., 2010), and only occasionally identified in *C. grandis* (Cheong et al., 2011a,b), *C. reticulata* (Pino and Quijano-Celís, 2007), *C. sinensis* (Dugo and Mondello, 2011), *C. aurantifolia* (Dugo and Mondello, 2011) and *C. medica* (Lota et al., 1999).

### Volatile Composition of *Citrus* Blossoms

There are fewer studies about the VOCs in the flowers of *Citrus* most common species in comparation with *Citrus* rind. These analyses have been mostly performed on *C. grandis, C. aurantium, C. limon, C. reticulata, C. sinensis, C. aurantifolia*, and *C. paradisi* blossoms. According to our review, many of the volatile compounds present in the *Citrus* peel have also been reported in *Citrus* blossom and neroli (Alissandrakis et al., 2003; Boussaada and Chemli, 2006; Jabalpurwala et al., 2009; Cheong et al., 2011b; Dugo et al., 2011; Sarrou et al., 2013; Družić, 2016). This is the case of many monoterpene hydrocarbons such as limonene, some monoterpene alcohol such as linalool and geraniol, some sesquiterpene hydrocarbons such as (*E)*-β-farnesene and β-caryophyllene, some alcohol sesquiterpene such as (*E*)-nerolidol and (*Z*,*E*)-farnesol, the norcarotenoid 6-methyl-5-hepten-2-one, and a few non-terpenoid aliphatic compounds such as hexanal. The common VOCs identified in the peel of all the 10 species described (Table 1) are not always reported in their corresponding blossoms. This is the case of the terpene compounds perillaldehyde, carvone, valencene or nootkatone (Supplementary Table S2). Interestingly, the non-terpenoid C_8_–C_12_ aliphatic aldehyde compounds, all of them common in the peel of all the revised species, have been sporadically described in flowers of some species as *C. paradisi* (Flamini and Cioni, 2010) or *C. grandis* (Huang et al., 2017).

Unlike in the peel EO, the most abundant compound in neroli is not always the monoterpene limonene, as it is sometimes replaced by linalool (Boussaada and Chemli, 2006; Sarrou et al., 2013). Moreover, according to Flamini and Cioni (2010) and Huang et al. (2017), *C. limon* and *C. grandis* neroli composition are different to those of whole flowers, because these neroli are richer in monoterpenes such as limonene, while their corresponding whole flowers are richer in alcohol monoterpenoids such as linalool. Moreover, some studies show that VOCs are produced in distinctive amounts by the different flower organs (Flamini et al., 2007; Flamini and Cioni, 2010; Huang et al., 2017).

In addition, there are some VOCs almost exclusively isolated in *Citrus* blossoms, which have been reported occasionally in *Citrus* peel and leaves, although they do not appear to be characteristic of any *Citrus* species in particular. Among them, we highlight nitrogenous compounds such as benzeneacetonitrile (Jabalpurwala et al., 2009; Flamini and Cioni, 2010; Družić, 2016), isolated in flowers of *C. reticulata, C. sinensis, C. aurantifolia, C. aurantium, C. paradisi* and *C. limon*; *N*-phenylformamide (Jabalpurwala et al., 2009), isolated in flowers of the same species except *C. limon*, or methyl anthranilate (Flamini et al., 2007; Jabalpurwala et al., 2009; Flamini and Cioni, 2010; Cheong et al., 2011b; Dugo et al., 2011; Sarrou et al., 2013; Ben Hsouna et al., 2017; Huang et al., 2017) and indole (Alissandrakis et al., 2003; Boussaada and Chemli, 2006; Boussaada et al., 2007; Flamini et al., 2007; Jabalpurwala et al., 2009; Flamini and Cioni, 2010; Zakaria et al., 2010; Družić, 2016; Ben Hsouna et al., 2017; Huang et al., 2017), identified in flowers of *C. grandis, C. limon, C. reticulata, C. sinensis, C. aurantium, C. aurantifolia*, and *C. paradisi*. Other compounds mainly detected in *Citrus* flowers are the aromatic compounds 2-phenylethanol [*C. grandis* (Cheong et al., 2011b), *C. reticulata* (Alissandrakis et al., 2003; Jabalpurwala et al., 2009), *C. paradisi* (Jabalpurwala et al., 2009; Flamini and Cioni, 2010), *C. aurantifolia* (Jabalpurwala et al., 2009), and *C. aurantium* (Jabalpurwala et al., 2009; Dugo et al., 2011; Družić, 2016)], benzaldehyde [*C. grandis* (Zakaria et al., 2010; Cheong et al., 2011b), *C. reticulata* (Jabalpurwala et al., 2009), *C. paradisi* (Jabalpurwala et al., 2009), *C. aurantifolia* (Jabalpurwala et al., 2009), *C. sinensis* (Jabalpurwala et al., 2009), *C. aurantium* (Družić, 2016), and *C. limon* (Jabalpurwala et al., 2009)], and phenylacetaldehyde [*C. grandis* (Cheong et al., 2011b), *C. reticulata* (Alissandrakis et al., 2003; Jabalpurwala et al., 2009), *C. sinensis* (Jabalpurwala et al., 2009), *C. aurantium* (Družić, 2016), and *C. limon* (Jabalpurwala et al., 2009)]. There is also a group of non-terpenoid aliphatic and olefinic compounds frequently reported in several *Citrus* flowers, but not in rinds or leaves, including acetone (Jabalpurwala et al., 2009; Cheong et al., 2011b), isopropanol (Jabalpurwala et al., 2009), (*Z*)-jasmone (Jabalpurwala et al., 2009; Flamini and Cioni, 2010; Dugo et al., 2011; Družić, 2016), or pentadecane (Jabalpurwala et al., 2009; Flamini and Cioni, 2010; Zakaria et al., 2010).

### Volatile Composition of *Citrus* Leaves

All the common VOCs reported in the peel of the 10 *Citrus* species studies (Table 1) have also been identified in the leaves of most of these species, except perillaldehyde, only reported in *C. limon* (Guerrini et al., 2014) (Supplementary Table S2). Almost all the terpenoid compounds described in *Citrus* peels have also been reported in leaves, except a few of them such as carvyl acetate, perillyl acetate, bornyl acetate or nootkatone. In contrast, only a few aromatic and nitrogen compounds have been described in *Citrus* leaves, with the exception of some compounds, such as methyl *N*-methylanthranilate in *C. reticulata* (Fanciullino et al., 2006; Tomi et al., 2008), *C. aurantium* (Kirbaslar and Kirbaslar, 2004; Dugo et al., 2011; Guerrini et al., 2014), *C. junos* (Huang et al., 2000) and *C. limon* (Kirbaslar and Kirbaslar, 2004). *Citrus* peel and leaves share many non-terpenoid aliphatic compounds, although some of them have not been reported in *Citrus* leaves, such as nonyl acetate, decyl acetate, dodecyl acetate or (*E,E)*-2,4-decadienal. At the same time, some non-terpenoid olefinic compounds are reported more frequently in *Citrus* leaves than in the peel, such as (*E*)-2-hexenal (Huang et al., 2000; Ahmed et al., 2001; Gancel et al., 2003, 2005) or (*Z*)-3-hexen-1-ol (Huang et al., 2000; Ahmed et al., 2001; Gancel et al., 2003, 2005), both with a distinctive grassy-green odor (Elmaci and Onogur, 2012). Even some compounds have only been found in leaves, such as (*Z*)-2-penten-1-ol (Gancel et al., 2003, 2005) or (*Z*)-3-hexenyl butanoate (Flamini and Cioni, 2010).

Recently a volatilome of *Citrus* leaves about differentially accumulated volatiles has been developed (Lamine et al., 2018). This study was performed in the leaves of four *Citrus* species (*C. limon*, *C. reticulata*, *C. sinensis* and *C. aurantium*) and these four species were clearly discriminated, especially *C. limon*. The number of taxonomic classifiers was narrow to eight (linalyl acetate, geranyl acetate, neryl acetate, β-*cis*-ocimene, δ-3-carene, β-myrcene, sabinene and α-terpineol). However, according to our review, it is very risky to establish taxonomic volatile metabolites with a single study, given the great variability of the volatile profile for the same species.

## Conclusion

This review about the volatiles in the *Citrus* essential oil summarized critically the volatile compounds identified up to date in the 10 most frequently studied *Citrus* species. It allowed their clustering based on the qualitative profile of the volatiles more reliably identified, which was in concordance with their genomic similitude. It also provides the possibility of developing species biomarkers based on this type of compounds, and points toward those which could be useful for the industry to monitor quality and consumer safety (Salgueiro et al., 2010). In addition, traceability, quality control, and adulterations of foods and drugs could be better addressed and optimized by using the *Citrus* volatile chemical database presented here. This review also provides important information to help in the selection of the most adequate species and the best source for specialized volatile chemicals for pharmaceutical, food, beverages, fragrances, and cosmetic industries.

## Author Contributions

MG-M reviewed the literature and elaborated the tables. JR performed statistical analysis and figures. ML-G elaborated Supplementary Figure S1. MG-M and JR drafted the manuscript. All authors listed have made a substantial, direct and intellectual contribution to the work, and approved it for publication.

## Conflict of Interest Statement

The authors declare that the research was conducted in the absence of any commercial or financial relationships that could be construed as a potential conflict of interest.

## Abbreviations

CAR: carboxen
DVB: divinylbenzene
EO: essential oil
FD: flavor dilution
GC-MS: gas chromatography-mass spectrometry
GC-O: gas chromatography-olfactometry
HCA: hierarchical cluster analysis
HS-SPME: headspace-solid phase microextraction
PDMS: polydimethylsiloxane
VOCs: volatile organic compounds
