# Supplementary material for: Volatile Compounds in Citrus Essential Oils: A Comprehensive Review
Source: Front Plant Sci. 2019 Feb 5;10:12. doi: 10.3389/fpls.2019.00012 (PMC6370709; doi:10.3389/fpls.2019.00012)
Supplement: Supplementary file 1 [file Table_1.DOCX]

**Supplementary Table 1. GC columns used to analyse volatile compounds of *Citrus* spp.**

| **Non-polar*** | **Polar*** |
| --- | --- |
| *^a^*HP-5, 60 x 0.25 (0.25)^1, 2^ | *^f^*HP-Innowax, 60 x 0.32 (0.5)^3^ |
| *^a^*HP-5MS, 60 x 0.25 (0.25)^4^ | *^f^*HP-Innowax, 60 x 0.25 (0.25)^5, 6^ |
| *^a^*RTx-5MS, 60 x 0.32 (0.25)^7, 8^ | *^f^*DB-Wax, 60 x 0.25 (0.25)^9-12^ |
| *^a^*DB-5, 30 x 0.25 (1)^13^ | *^f^*InertCap Wax, 60 x 0.25 (0.25)^14, 15^ |
| *^a^*OPTIMA-5-MS, 30 x 0.25 (0.50)^16^ | *^f^*Rtx-Wax, 60 x 0.32 (0.25)^7, 8^ |
| *^a^*DB-5, 30 x 0.25 (0.33)^17^ | *^f^*BC-Wax, 50 x 0.25 (0.15)^18^ |
| *^a^*HP-5MS, 30 x 0.25 (0.32)^19^ | *^f^*DB-Wax, 30 x 0.25 (0.25)^20^ |
| *^a^*DB-5, 30 x 0.25 (0.25)^21-27^ | *^f^*HP-Innowax, 30 x 0.25 (0.25)^28^ |
| *^b^*Elite 5-MS, 30 x 0.25 (0.25)^29^ | *^f^*SupelcoWax 10, 30 x 0.25 (0.25)^30, 31^ |
| *^a^*VF5-MS, 30 x 0.25 (0.25)^32^ | *^f^*HP-Wax, 30 m x 0.25 (0.25)^33^ |
| *^a^*TRACE TR-5 MS, 30 x 0.25 (0.25)^34-36^ | *^f^*DB-Wax, 30 x 0.32 (0.25)^37^ |
| *^a^*HP-5MS, 30 x 0.25 (0.25)^28, 38-50^ | *^g^*DB-FFAP, 60 x 0.25 (0.25)^51, 52^ |
| *^a^*DM-5MS, 30 x 0.25 (0.25)^53^ | *^h^*SLB-IL59, 30 x 0.25 (0.20)^49^ |
| *^c^*SLB-5MS, 30 x 0.25 (0.25)^54, 55^ |  |
| *^a^*MDN-5, 30 x 0.25 (0.25 µm)^56^ |  |
| *^a^*VF-5MS, 30 x 0.25 (0.15 µm)^57^ |  |
| *^a^*DB-5, 30 x 0.32 (0.5)^20^ |  |
| *^a^*DB-5, 30 x 0.32 (0.25)^37^ |  |
| *^d^*DB-1, 60 x 0.25 (0.25)^58, 59^ |  |
| *^d^*Rtx-1, 60 x 0.22 (0.25)^60, 61^ |  |
| *^d^*SPB-1, 30 x 0.25 (1)^30, 31^ |  |
| *^d^*DB-1, 30 x 0.25 (0.25)^58, 62^ |  |
| *^d^*HP-1, 30 x 0.25 (0.25)^63^ |  |
| *^d^*Zebron Z-1, 30 x 0.32 (0.25)^64^ |  |
| *^e^*PONA, 50 x 0.2 mm (0.5)^65^ |  |

*Length in m x intern diameter in mm (μm ﬁlm thickness). Phases: *^a^*5% diphenyl/95% dimethyl polysiloxane; *^b^*1,4-bis(dimethylsiloxy)phenylene dimethyl polysiloxane; *^c^*silphenylene polymer; *^d^*100% dimethylpolysiloxane; *^e^*crosslinked methylsilicone gum; *^f^*polyethylene glycol; *^g^*polyethyleneglycol modified with nitroterephthalic acid; *^h^*Non-bonded, 1,12-di(tripropylphosphonium) dodecanebis-(trifluoromethane-sulfonyl)imide.

**References for Supplementary Table 1**

1. Hosni, K., Zahed, N., Chrif, R., Abid, I., Medfei, W., Kallel, M., et al. (2010). Composition of peel essential oils from four selected Tunisian Citrus species: Evidence for the genotypic influence. *Food Chem. 123*, 1098-1104.

2. Jirapakkul, W., Tinchan, P., and Chaiseri, S. (2013). Effect of drying temperature on key odourants in kaffir lime (*Citrus hystrix* D.C., Rutaceae) leaves. *Int. J. Food Sci. Tech.* *48*, 143-149.

3. Dong, Z. B., Shao, W. Y., and Liang, Y. R. (2014). Isolation and characterization of essential oil extracted from tangerine peel. *Asian J. Chem. 26* (16), 4975-4978.

4. Hai Dang, N., Huong Nhung, P., Mai Anh, B. T., Thu Thuy, D. T., Van Minh, C., Tien Dat, N. (2016). Chemical composition and a-glucosidase inhibitory activity of Vietnamese *Citrus* peels essential oils. *J. Chem.* http://dx.doi.org/10.1155/2016/6787952.

5. Xiao, Z., Ma, S., Niu, Y., Chen, F., and Yu, D. (2016). Characterization of odour-active compounds of sweet orange essential oils of different regions by gas chromatography-mass spectrometry, gas chromatography-olfactometry and their correlation with sensory attributes. *Flavour Frag. J.* *31*, 41-50.

6. Miah, M. N., Bachar, S. C., Nahar, L., Rhaman, M. S., Rashid, M. A., Hadiuzzaman, S., et al. (2010). Composition of the volatiles of *Citrus macroptera* var. *annamensis* and evaluation of bioactivity. *J. Essent. Oil Bear. Pl.* *13* (2), 211-218.

7. Sun, H., Ni, H., Yang, Y., Wu, L., Cai, H.-N., Xiao, A.-F., et al. (2014). Investigation of sunlight-induced deterioration of aroma of pummelo (*Citrus maxima*) essential oil. *J. Agric. Food Chem.* *62*, 11818-11830.

8. Li, L. J., Hong, P., Chen, F., Sun, H., Yang, Y. F., Yu, X., et al. (2016). Characterization of the aldehydes and their transformations induced by uv irradiation and air exposure of white guanxi honey pummelo (*Citrus grandis* (L.) Osbeck) essential oil. *J. Agric. Food Chem.* *64*, 5000-5010.

9. Akakabe, Y., Kusunoki, A., Tanaka, R., and Kanetsune, Y. (2010). A comparison of volatile components of Setomi with its parent cultivars. *Biosci. Biotechnol. Biochem. 74* (3), 659-662.

10. Omori, H., Nakahara, K., and Umano, K. (2011). Characterization of aroma compounds in the peel extract of Jabara (*Citrus jabara* Hort. ex Tanaka). *Flavour Frag. J. 26*, 396-402.

11. Asikin, Y., Maeda, G., Tamaki, H., Mizu, M., Oku, H., and Wada, K. (2015). Cultivation line and fruit ripening discriminations of Shiikuwasha (*Citrus depressa* Hayata) peel oils using aroma compositional, electronic nose, and antioxidant anlyses. *Food Res. Int.* *67*, 102-110.

12. Asikin, Y., Taira, I., Inafuku, S., Sumi, H., Sawamura, M., Takara, K., et al. (2012). Volatile aroma components and antioxidant activities of the flavedo peel extract of unripe shiikuwasha (*Citrus depressa* Hayata). *J. Food Sci.* *77* (4), 469-475.

13. Lin, S. Y., Roan, S. F., Lee, C. L., and Chen, I. Z. (2010). Volatile organic components of fresh leaves as indicators of indigenous and cultivated citrus species in Taiwan. *Biosci. Biotechnol. Biochem.* *74* (4), 806-811.

14. Miyazato, H., Hashimoto, S., and Hayashi, S. (2013). First identification of the odour-active unsaturated aliphatic acid (*E*)-4-methyl-3-hexenoic acid in yuzu (*Citrus junos* Sieb. ex Tanaka). *Flavour Frag. J.* *28*, 62-69.

15. Miyazato, H., Hashimoto, S., and Hayashi, S. (2012). Identification of the odour-active aldehyde *trans*-4,5-epoxy-(*E,Z*)-2,7-decadienal in yuzu (*Citrus junos* Sieb. ex Tanaka). *Eur. Food Res. Technol*, *235* (5), 881-891.

16. Ndiaye, E. H. B., Talla Gueye, M., Ndiaye, I., Mbacké Diop, S., Bakar Diop, M., Thiam, A., et al. (2017). Chemical composition of distilled essential oils and hydrosols of four Senegalese Citrus and enantiomeric characterization of chiral compounds. *J. Essent. Oil Bear. Pl.* **2017,** *20* (3), 820-834.

17. Aliberti, L., Caputo, l., de Feo, V., de Martino, L., Nazzaro, F., Souza, L. F. (2016). Chemical composition and in vitro antimicrobial, cytotoxic, and central nervous system activities of the essential oils of *Citrus medica* L. cv. ‘Liscia’ and *C. medica* cv. ‘Rugosa’ cultivated in Southern Italy. *Molecules*  *21*, 1244, doi:10.3390/molecules21091244

18. Tomiyama, K., Aoki, H., Oikawa, T., Sakurai, K., Kashara, Y., and Kawakami, Y. (2012). Characteristic volatile components of Japanese sour citrus fruits: Yuzu, Sudachi and Kabosu. *Flavour Frag. J.* *27*, 341-355.

19. Yang, S.-A., Jeon, S.-K., Lee, E.-J., Shim, C.-H., and Lee, I.-S. (2010). Comparative study of the chemical composition and antioxidant activity of six essential oils and their components. *Nat. Prod. Res. 24* (2), 140-151.

20. Deterre, S., Rega, B., Delarue, J., Decloux, M., Lebrun, M., and Giampaoli, P. (2012). Identification of key aroma compounds from bitter orange (*Citrus aurantium* L.) products: essential oil and macerate-distillate extract. *Flavour Frag. J.* *27*, 77-88.

21. Darjazi, B. B. (2011). Comparison of volatile components of flower, leaf, peel and juice of 'Page' mandarin [(*Citrus reticulata* var '*Dancy*' x *Citrus paradisi* var '*Duncan*') x *Citrus clementina*]. *Afr. J. Biotechnol. 10* (51), 10437-10446.

22. Jiang, M.-H., Yang, L., Zhu, L., Piao, J.-H., and Jiang, J.-G. (2011). Comparative GC/MS analysis of essential oils extracted by 3 methods from the bud of *Citrus aurantium* L. var. *amara* Engl. *J. Food Sci.* *76* (9), 1219-1225.

23. Kerdchoechuen, O., Laohakunjit, N., Singkornard, S., and Matta, F. B. (2010). Essential oils from six herbal plants for biocontrol of the maixe weevil. *HortScience 45* (4), 592-598.

24. Darjazi, B. B. (2011). The effects of rootstock on the volatile flavor components of page mandarin [(*Citrus reticulata* var dancy x *Citrus paradisi* var dancan) x *Citrus clementina*] flower and leaf. *Afr. J. Agric. Res.* *6*, 1884-1896.

25. Darjazi, B. B. (2011). A comparison of volatile components of flower of page mandarin obtained by ultrasound-assisted extraction and hydrodistillation. *J. Med. Plants Res. 5* (13), 2839-2847.

26. Ellouze, I., Abderrabba, M., Sabaou, N., Mathieu, F., Lebrihi, A., and Bouajila, J. (2012). Season's variation impact on *Citrus aurantium* leaves essential oil: Chemical composition and biological activities. *J. Food Sci.* *77* (9), 173-180.

27. Chung, H., Chung, W.-Y., Yoo, E.-S., Cho, S. K., Oh, S.-K., and Kim, Y.-S. (2012). Characterization of volatile aroma-active compounds in Dangyooja (*Citrus grandis* Osbeck). *J. Korean Soc. Appl. Biol. Chem. 55*, 133-136.

28. Jabri Karoui, I., and Marzouk, B. (2013). Characterization of bioactive compounds in Tunisian bitter orange (*Citrus aurantium L*.) peel and juice and determination of their antioxidant activities. *Biomed. Res. Int.* <http://dx.doi.org/10.1155/2013/345415>.

29. Killiny, N., and Jones, S. E. (2017). Proﬁling of volatile organic compounds released from individual intact juvenile and mature citrus leaves. *J. Plant Physiol.* *208*, 47-51.

30. Delort, E., Jaquier, A., Decorzant, E., Chapuis, C., Casilli, A., and Frérot, E. (2015). Comparative analysis of three Australian finger lime (*Citrus australasica*) cultivars: Identification of unique Citrus chemotypes and new volatile molecules. *Phytochemistry* *109*, 111-124.

31. Casilli, A., Decorzant, E., Jaquier, A., and Delort, E. (2014). Multidimensional gas chromatography hyphenated to mass spectrometry and olfactometry for the volatile analysis of Citrus hybrid peel extract. *‎J. Chromatogr. A* *1373*, 169-178.

32. Benelli, P., Riehl, C. A. S., Smânia, J. A., Smânia, E. F. A., and Ferreira, S. R. S. (2010). Bioactive extracts of orange (*Citrus sinensis* L. Osbeck) pomace obtained by SFE and low pressure techniques: Mathematical modeling and extract composition. *J. Supercrit. Fluids* *55*, 132-141.

33. Chen, Y., Wu, J., Xu, Y., Fu, M., and Xiao, G. (2014). Effect of second cooling on the chemical components of essential oils from orange peel (*Citrus sinensis)*. *J. Agric. Food Chem.* *62*, 8786-8790.

34. Liu, C., Cheng, Y., Zhang, H., Deng, X., Chen, F., and Xu, J. (2012). Volatile constituents of wild Citrus Mangshanyegan (*Citrus nobilis* Lauriro) peel oil. *J. Agric. Food Chem.* **2012,** *60*, 2617-2628.

35. Liu, C., Yan, F., Gao, H., He, M., Wang, Z., Cheng, Y., et al. (2015). Features of citrus terpenoid production as revealed by carotenoid, limonoid and aroma profiles of two pummelos (*Citrus maxima*) with different flesh color. *J. Sci. Food Agric. 95*, 111-119.

36. Zhang, H., Xie, Y., Liu, C., Chen, S., Hu, S., Xie, Z., et al. (2017). Comprehensive comparative analysis of volatile compounds in citrus fruits of different species. *Food Chem. 230*, 316-326.

37. Inafuku-Teramoto, S., Suwa, R., Fukuzawa, Y., and Kawamitsu, Y. (2011). Polymethoxyflavones, synephrine and volatile constitution of peels of citrus fruit grown in Okinawa. *J. Jpn. Soc. Hortic. Sci.* *80* (2), 214-224.

38. Zakaria, Z., Zakaria, S., and Ishak, M. Z. M. (2010). Analysis of major fragrant compounds from *Citrus grandis* flowers extracts. *Sains Malays. 39* (4), 565-569.

39. Wang, H., Tao, N., Huang, S., and Liu, Y. (2012). Effect of shatangju (*Citrus reticulata* blanco) essential oil on spore germination and mycelium growth of *Penicillium digitatum* and *P. italicum*. *J. Essent. Oil Bear. Pl. 15* (5), 715-723.

40. Tao, N.-G., and Liu, Y.-J. (2012). Chemical composition and antimicrobial activity of the essential oil from the peel of Shatian pummelo (*Citrus grandis* Osbeck). *Int. J. Food Prop.* *15*, 709-716.

41. Liu, K., Che, Q., Liu, Y., Zhou, X., and Wang, X. (2012). Isolation and biological activities of decanal, linalool, valencene, and octanal from sweet orange oil. *J. Food Sci. 77* (11), 1156-1161.

42. Chung, M. S. (2012). Volatile compounds of the *Hallabong* (*Citrus kiyomi x* *Citrus ponkan*) blossom. *Food Sci. Biotechnol. 21* (1), 285-290.

43. Bourgou, S., Rahali, F. Z., Ourghemmi, I., Tounsi, M. S. (2012). Changes of peel essential oil composition of four Tunisian *Citrus* during fruit maturation. *Sci. World J.* doi: 10.1100/2012/528593.

44. Elmaci, Y., and Onogur, T. A. (2012). Mandarin peel aroma: Estimation by using headspace/GC/MS and descriptive analysis techniques. *Acta Aliment. 41* (1), 131-139.

45. Wang, J., and Liu, Y. (2014). Comparative study on the volatiles in *Citrus reticulata* 'Dahongpao' peel from the same plant and their antioxidant activities. *J. Essent. Oil Bear. Pl.* *17* (2), 303-308.

46. Luciardi, M. C., Blázquez, M. A., Cartagena, E., Bardón, A., and Arena, M. E. (2016). Mandarin essential oils inhibit quorum sensing and virulence factors of *Pseudomonas aeruginosa*. *LWT-Food Sci. Technol.* *68*, 373-380.

47. Blázquez, M. A., and Carbó, E. (2015). Control of *Portulaca oleracea* by boldo and lemon essential oils in different soils. *Ind. Crops Prod.* *76*, 515-521.

48. Tao, N., Jia, L., and Zhou, H. (2014). Anti-fungal activity of *Citrus reticulata* Blanco essential oil against *Penicillium italicum* and *Penicillium digitatum*. *Food Chem. 153*, 265-271.

49. Ragonese, C., Sciarrone, D., Tranchida, P. Q., Dugo, P., Dugo, G., and Mondello, L. (2011). Evaluation of a medium-polarity ionic liquid stationary phase in the analysis of flavor and fragrance compounds. *Anal. Chem.* *83*, 7947-7954.

50. Loizzo, M. R., Tundis, R., Bonesi, M., Di Sanzo, G., Verardi, A., Lopresto, C. G., et al. (2016). Chemical profile and antioxidant properties of extracts and essential oils from *Citrus x limon* (L.) BURM. cv. Femminello comune. *Chem. Biodiversity* *13*, 571-581.

51. Cheong, M. W., Loke, X.-Q., Liu, S.-Q., Pramudya, K., Curran, P., and Yu, B. (2011). Characterization of volatile compounds and aroma profiles of Malaysian pomelo (*Citrus grandis* (L.) *Osbeck*) blossom and peel. *J. Essent. Oil Res.* *23*, 33-44.

52. Cheong, M. W., Chong, Z. S., Liu, S.-Q., Zhou, W., Curran, P., and Yu, B. (2012). Characterization of calamansi (*Citrus microcarpa*). Part I: Volatiles, aromatic profiles and phenolic acids in the peel. *Food Chem. 134*, 686-695.

53. Wu, Z., Li, H., Yang, Y., Zhan, Y., and Tu, D. (2013). Variation in the components and antioxidant activity of *Citrus medica* L. var. *sarcodactylis* essential oils at different stage of maturity. *Ind. Crops Prod.* *46*, 311-316.

54. Spadaro, F., Circosta, C., Costa, R., Pizzimenti, F., Palumbo, D. R., and Occhiuto, F. (2012). Volatile fraction composition and biological activity of lemon oil (*Citrus limon* L. Burm.): Comparative study of oils extracted from conventionally grown and biological fruits. *J. Essent. Oil Res.* *24* (2), 187-193.

55. Costa, R., Bisignano, C., Filocamo, A., Grasso, E., Occhiuto, F., and Spadaro, F. (2014). Antimicrobial activity and chemical composition of *Citrus aurantifolia* (Christm.) Swingle essential oil from Italian organic crops. *J. Essent. Oil Res.* *26* (6), 400-408.

56. Furneri, P. M., Mondello, L., Mandalari, G., Paolino, D., Dugo, P., Garozzo, A., et al. (2012). *In vitro* antimycoplasmal activity of *Citrus bergamia* essential oil and its major components. *Eur. J. Med. Chem.* *52*, 66-69.

57. Guerrini, A., Rossi, D., Grandini, A., Scalvenzi, L., Noriega Rivera, P. F., Andreotti, E., et al. (2014). Biological and chemo-diverse characterization of Amazonian (Ecuador) *Citrus* petitgrains. *J. Appl. Bot. Food Qual.* *87*, 108-116.

58. Pashazanousi, M. B., Raeesi, M., and Shirali, S. (2012). Chemical composition of the essential oil, antibacterial and antioxidant activities, total phenolic an flavonoid evaluation of various extracts from leaves and fruit peels of *Citrus limon*. *Asian J. Chem. 24* (10), 4331-4334.

59. Torres-Alvarez, C., Núñez González, A., Rodríguez, J., Castillo, S., Leos-Rivas, C., and Báez-González, J. G. (2017). Chemical composition, antimicrobial, and antioxidant activities of orange essential oil and its concentrated oils. *CyTA J. Food 15* (1), 129-135.

60. Paoli, M., de Rocca Serra, D., Tomi, F., Luro, F., and Bighelli, A. (2016). Chemical composition of the leaf essential oil of grapefruits (*Citrus paradisi* Macf.) in relation with the genetic origin. *J. Essent. Oil Res.* *28* (4), 265-271.

61. Luro, F., Venturini, N., Costantino, G., Paolini, J., Ollitrault, P., and Costa, J. (2012). Genetic and chemical diversity of citron (*Citrus medica* L.) based on nuclear and cytoplasmic markers and leaf essential oil composition. *Phytochemistry* *77*, 186-196.

62. Sultana, H. S., Ali, M., and Panda, B. P. (2012). Influence of volatile constituents of fruit peels of *Citrus reticulata Blanco* on clinically isolated pathogenic microorganisms under *In-vitro*. *Asian J. Chem.* S1299-S1302.

63. Chintaluri, A. K., Komarraju, A. L., Chintaluri, V. K., and Vemulapalli, B. (2015). Comparative study and antimicrobial activity of essential oils of selected plants of Rutaceae and TLC bioautographic studies for detection of bioactive compounds. *J. Essent. Oil Res.* *27* (1), 9-16.

64. Velázquez-Nuñez, M. J., Avila-Sosa, R., Palou, E., and López-Malo, A. (2013). Antifungal activity of orange (*Citrus sinensis* var. Valencia) peel essential oil applied by direct addition or vapor contact. *Food Control* *31*, 1-4.

65. Sokovic, M., and van Griensven, L. J. L. D. (2006). Antimicrobial activity of essential oils and their components against the three major pathogens of the cultivated button mushroom, *Agaricus bisporus*. *Eur. J. Plant Pathol.* *116*, 211-224.
