# Supplementary material for: Volatile Compounds in Citrus Essential Oils: A Comprehensive Review
Source: Front Plant Sci. 2019 Feb 5;10:12. doi: 10.3389/fpls.2019.00012 (PMC6370709; doi:10.3389/fpls.2019.00012)

Supplementary Figure 1 (Figure S1). Chemical structures of the terpenoid compounds identified in all ten most frequently studied *Citrus* species.

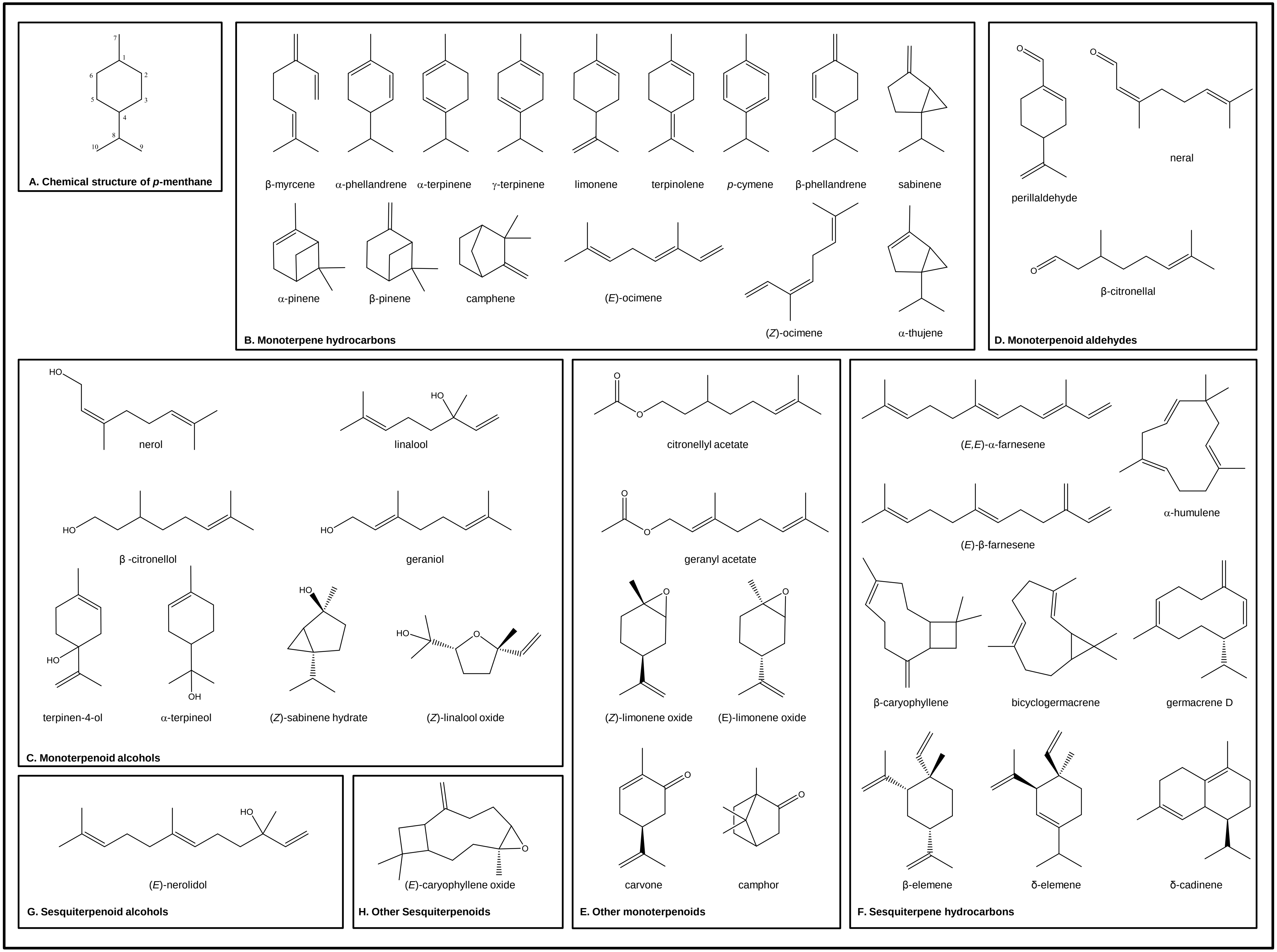

Supplement: Supplementary file 6 [file Data_Sheet_1.pdf]
